# AI-enabled routine H&E image based prognostic marker for early-stage luminal breast cancer

**DOI:** 10.1038/s41698-023-00472-y

**Published:** 2023-11-15

**Authors:** Noorul Wahab, Michael Toss, Islam M. Miligy, Mostafa Jahanifar, Nehal M. Atallah, Wenqi Lu, Simon Graham, Mohsin Bilal, Abhir Bhalerao, Ayat G. Lashen, Shorouk Makhlouf, Asmaa Y. Ibrahim, David Snead, Fayyaz Minhas, Shan E. Ahmed Raza, Emad Rakha, Nasir Rajpoot

**Affiliations:** 1 Tissue Image Analytics Centre, Department of Computer Science, University of Warwick, Coventry, UK; 2 Academic Unit for Translational Medical Sciences, School of Medicine, University of Nottingham, Nottingham, UK; 3 Department of Histopathology, Sheffield Teaching Hospitals NHS Trust, Sheffield, UK; 4 Department of Pathology, Faculty of Medicine, Menoufia University, Shebin El-Koum, Egypt; 5 Histofy Ltd, Birmingham, UK; 6 Department of Pathology, Faculty of Medicine, Assiut University, Asyut, Egypt; 7 The Alan Turing Institute, London, UK

**Keywords:** Prognostic markers, Cancer

## Abstract

Breast cancer (BC) grade is a well-established subjective prognostic indicator of tumour aggressiveness. Tumour heterogeneity and subjective assessment result in high degree of variability among observers in BC grading. Here we propose an objective Haematoxylin & Eosin (H&E) image-based prognostic marker for early-stage luminal/Her2-negative **BR**e**A**st **C**anc**E**r that we term as the BRACE marker. The proposed BRACE marker is derived from AI based assessment of heterogeneity in BC at a detailed level using the power of deep learning. The prognostic ability of the marker is validated in two well-annotated cohorts (Cohort-A/Nottingham: *n* = 2122 and Cohort-B/Coventry: *n* = 311) on early-stage luminal/HER2-negative BC patients treated with endocrine therapy and with long-term follow-up. The BRACE marker is able to stratify patients for both distant metastasis free survival (*p* = 0.001, C-index: 0.73) and BC specific survival (*p* < 0.0001, C-index: 0.84) showing comparable prediction accuracy to Nottingham Prognostic Index and Magee scores, which are both derived from manual histopathological assessment, to identify luminal BC patients that may be likely to benefit from adjuvant chemotherapy.

## Introduction

Breast cancer (BC) is the most common cancer in women with an estimated 2.3 million cases and 0.7 million deaths reported worldwide in 2020^1^. BC is a heterogeneous disease with different molecular subtypes, variable morphology presentation, behaviour and response to therapy^2,3^. With the introduction of endocrine therapy, the prognosis of early-stage oestrogen receptor positive (ER+) and human epidermal growth factor receptor 2 negative (HER2−) BC, which comprises approximately 40% of BC^4^, has improved^5^ but in about 20% of the cases the decease can still recur post-treatment^6^. Because some of the patients in this early-stage luminal BC will benefit from adjuvant chemotherapy while others will only require endocrine therapy, it is important to risk-stratify these patients for better treatment management^7,8^. Stratification of patients into risk groups based on their survival outcome is key for personalised treatment and therapeutic interventions and, therefore, identification of clinicopathological factors and biomarkers is important area of clinical research^9,10^.

Despite several advancements in BC diagnosis and management, the existing risk stratification tools are subjective and unable to cope with the highly heterogenous morphology of the BC histology. Current BC management relies on the availability of robust clinical and pathological prognostic factors to support clinical management decision making. The Nottingham grading system (NGS)^11^, which comprises the assessment of three morphological features (tubule formation, mitotic count and nuclear pleomorphism), is a well-established prognostic marker in BC that is recommended by the World Health Organisation and other national and international organisations^12,13^ as the gold standard BC grading system. NGS is a simple and cost-effective prognostic tool that was recently incorporated into the tumour-node-metastasis (TNM) stage system (prognostic stage)^14^. However, NGS still relies on subjective assessment of histology samples which needs to be resolved for reproducible, robust and reliable patient stratification.

Though NGC being an established prognostic marker its performance in predicting the outcome of the clinically indeterminate group of early-stage luminal BC is still non-optimal. Reproducibility concerns have been raised due to inter-observer disagreement regarding grade components and the complexity of intra-tumour heterogeneity^15,16^. Therefore, molecular tests including multigene assays such as Oncotype DX(ODX)^17^ and PAM50^18^ are increasingly used to risk stratify this group of BC. However, the relatively high cost and turnaround time and the relatively low concordance between assays makes the development of objective, reproducible and reliable alternative methods such as AI-based prognostic tools highly warranted.

Deep learning (DL) based analysis of haematoxylin & eosin (H&E) stained histology scanned slide has produced remarkable results for objective assessment of morphological features^19–26^. Several studies^27–31^ have adapted DL for survival analysis, including an ensemble of deep convolutional neural network (CNN) models for risk stratification of BC^32^. Recently, other studies^33,34^ have been focused on the importance of developing image-based tools to risk stratify the clinically indeterminate risk class of BC. The correlation of ODX and mitotic count has been previously demonstrated in different studies^35,36^ whereas some studies have reported a combination of image-based features with clinicopathological variables^37,38^. Though some studies^36,39^ have shown that different structures, tissue types and cells types have prognostic importance but a comprehensive phenotyping of such structures and their relationship with survival outcome is less studied especially for luminal early stage BC.

**Fig. 1:**
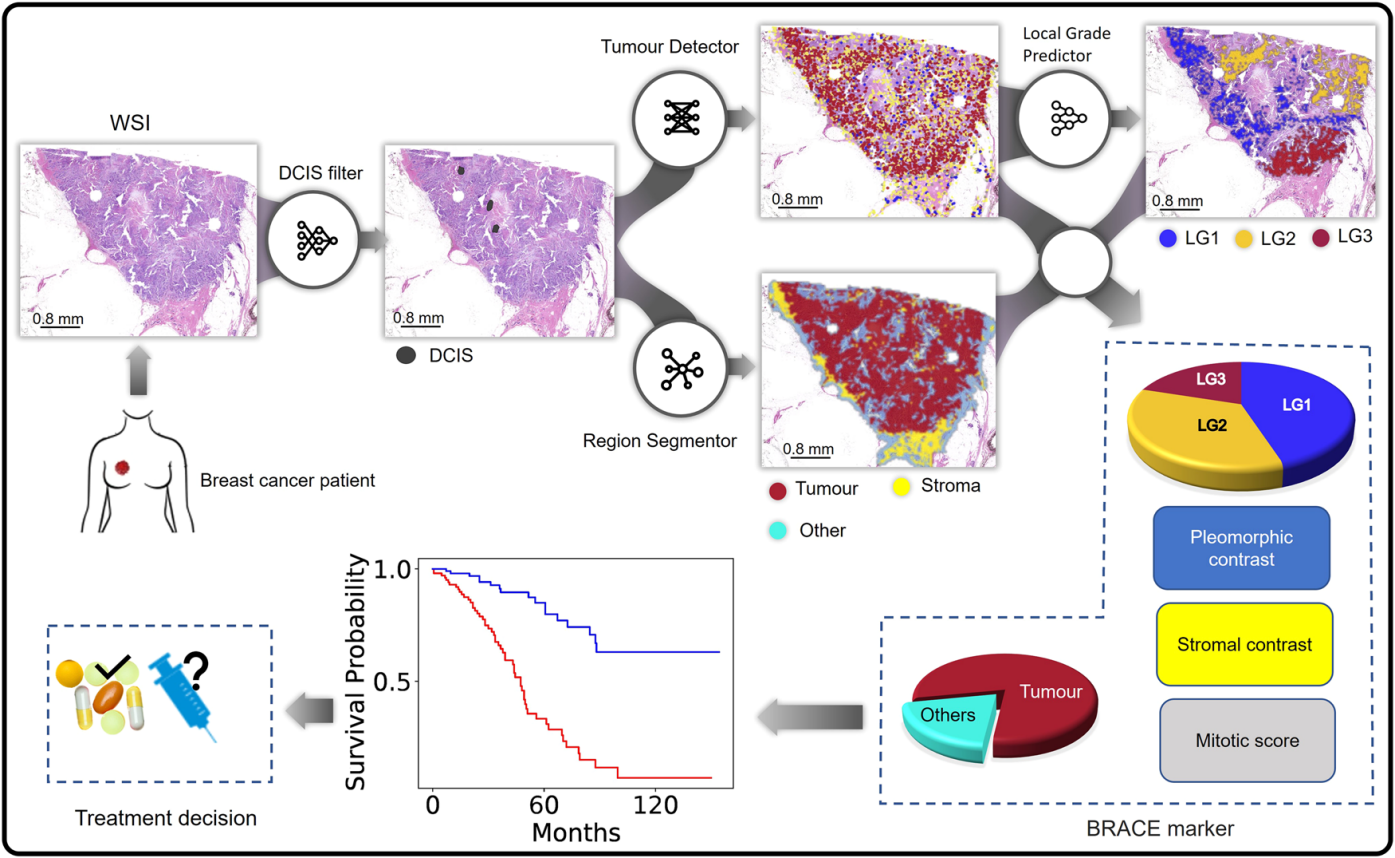
Proposed BRACE marker workflow for breast cancer survival prediction. DCIS regions are filtered for exclusion in the following steps; Tumour Detector segments and classify nuclei for tumour rich area identification/ROI selection; prediction of local grade composition (digital local grade LG1–3) and pleomorphism by Local Grade Predictor which is trained in a supervised way by using clinical grade as WSI-level label; Tissue region segmentation by Region Segmentor to quantify stromal cell density and tumour area percentage; Using the extracted features to form BRACE marker for survival prediction.

In this study, we show that the DL-based automated phenotyping of BC can provide a cost-effective and reproducible prognostic tool. Using whole slide images (WSIs) of a large well characterised cohort of luminal early stage BC, we develop an AI-based BRACE marker to capture tumour and stromal heterogeneity and mitotic activity in a quantitative and reproducible manner (Fig. 1). In addition to objectively quantifying the stromal and pleomorphic heterogeneity and digital mitotic score, the proposed marker utilises the spatial composition of digital local tumour grading (Tumour Grade Composition (TGC)) as opposed to a single case-level grade. The method is validated for prognostication of early-stage luminal breast cancer on an external cohort. Development of such AI-based markers will provide new research alternatives leading to integrated solutions along with gene expression profiling.

## Results

To estimate the BRACE marker first the ductal carcinoma in situ (DCIS) regions were filtered. Second, tumour rich areas were identified from tumour cell detections. This step identified regions of interest (ROI) for digital mitotic counting as well as predicting local tumour grades and nuclear pleomorphism in the tumour rich areas. Third, tissue regions were segmented to quantify stromal cell density and tumour area percentage. Finally, the tumour grade composition, local variations in nuclear pleomorphism and stroma cell density, digital mitotic count and tumour area percentage were used for survival prediction (Fig. 1). Note that tissue area was selected by thresholding but this step is not shown in the figure to keep it simple.

### AI-based WSI phenotyping

Two well-characterised retrospective cohorts (Supplementary Table 1) with endocrine therapy were used for model construction for analysis of two endpoints i.e. distant metastasis free survival (DMFS) and BC specific survival (BCSS), with data splits detailed in supplementary Fig. 1. BC WSI contains a rich heterogeneous phenotype including tumour morphology, stromal variations, mitotic activity, tumour infiltrating lymphocytes (TILs) etc. The power of AI was utilised to explore a range of features (Supplementary Table 2) related to six main categories: (a) Tumour morphology (grade, pleomorphism), (b) tumour-stroma relationship, (c) TILs quantification, (d) heterogeneity in terms of tumour, stroma and TILs, (e) mitotic cell counting, and (f) counts/ratios of different nuclei. Following feature selection (see ‘Feature selection’ for details), the final features included: digital local grade composition in the form of local grade 1 percentage (LG1 %), LG2 %, LG3 %, tumour area %, pleomorphic contrast, stromal contrast, co-occurrences of stromal nuclei patches with low density, and digital mitotic score.

**Fig. 2:**
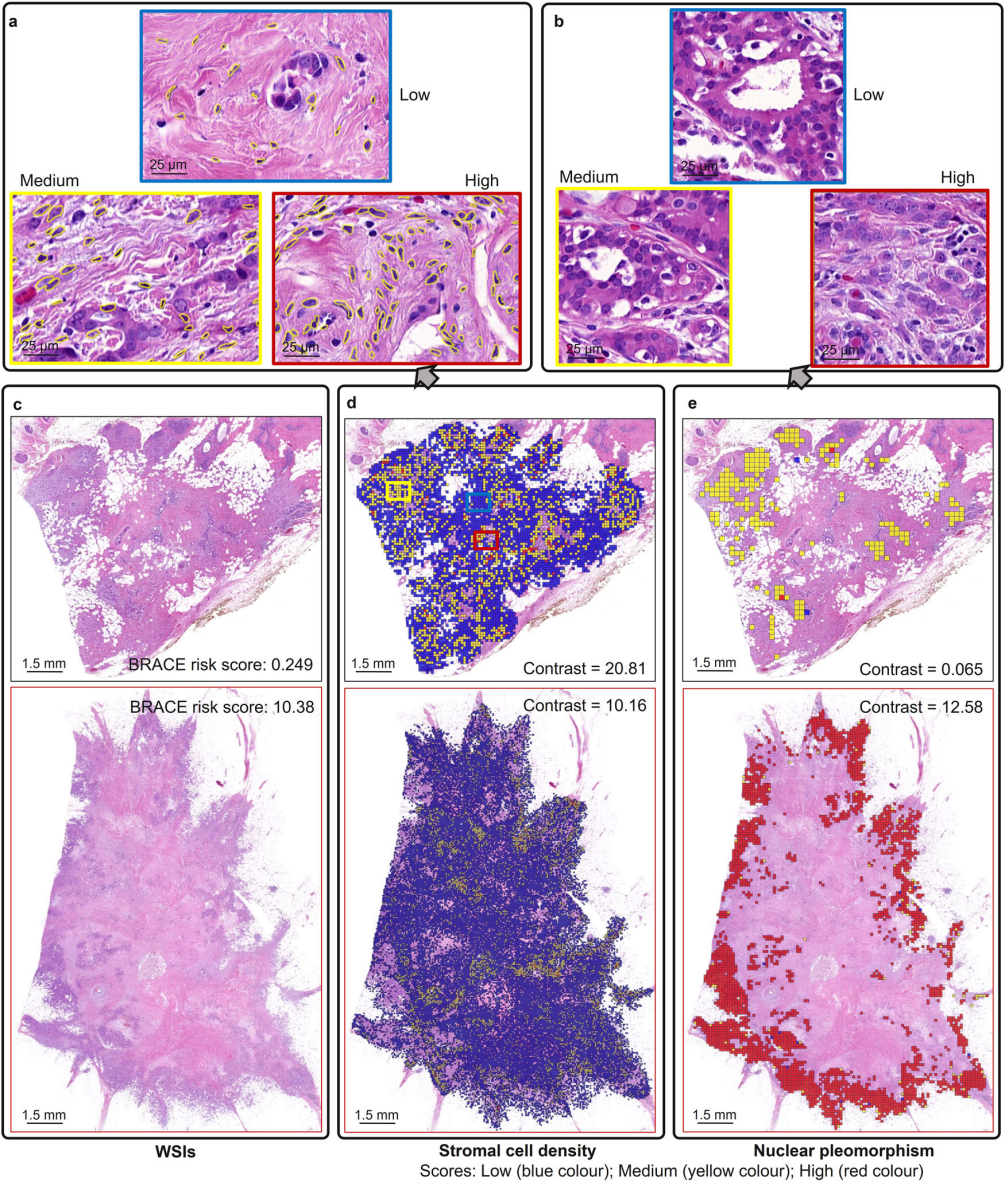
Stromal contrast and nuclear pleomorphism features. **a** Sample patches/areas of low, medium and high stomal cell density used to calculate stromal contrast in (**d**). **b** sample patches of low, medium and high nuclear pleomorphism used to calculate pleomorphic contrast in (**e**). **c** Two WSIs with their corresponding BRACE risk scores. **d** Stromal cell density for calculation of stromal contrast. **e** Nuclear pleomorphism used for calculation of pleomorphic contrast.

**Fig. 3:**
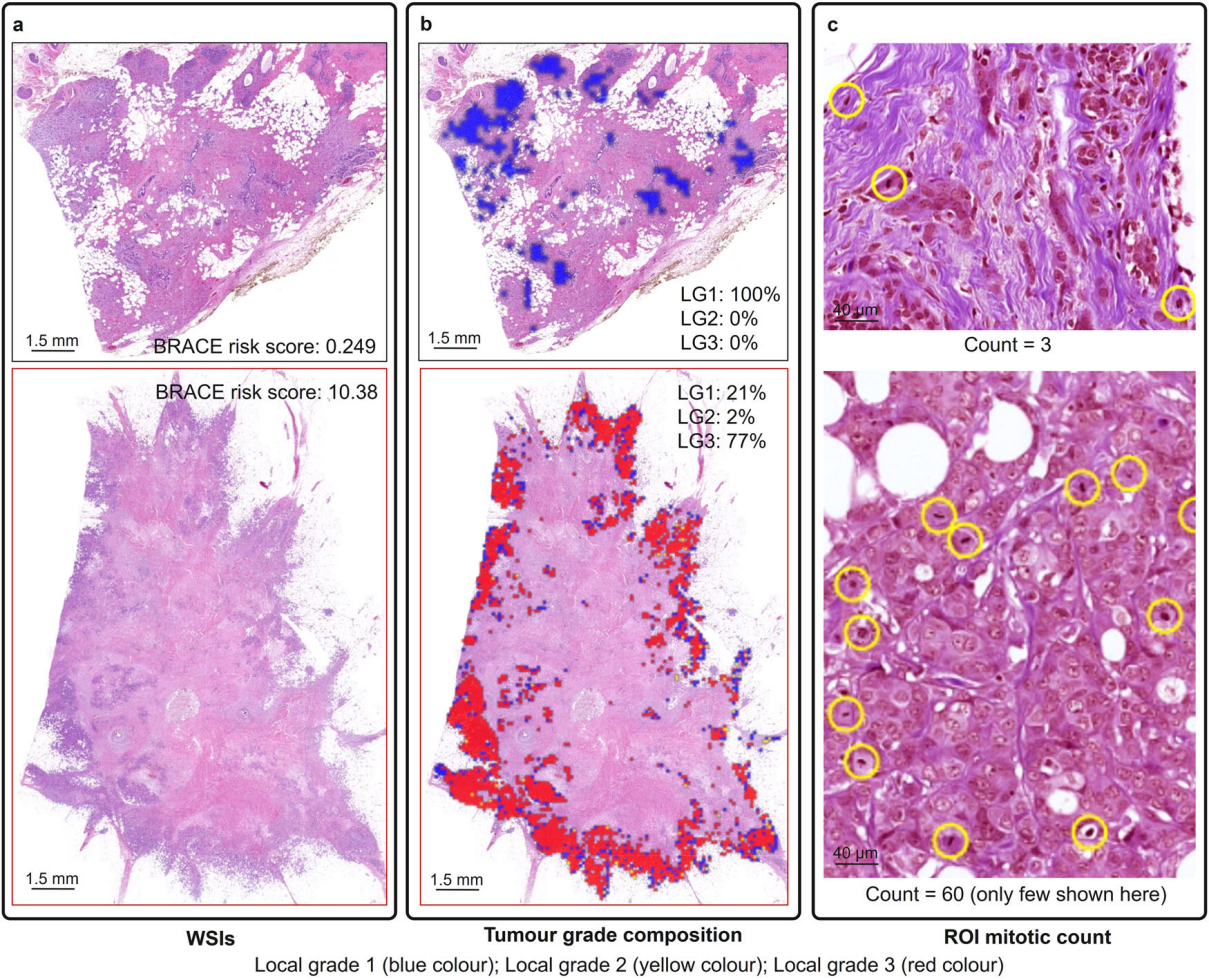
Tumour grade composition and mitotic counting features. **a** Two WSIs with their corresponding BRACE risk scores. **b** digital tumour grade composition showing percentage of each grade in a WSI. **c** Mitotic counts in a sample area extracted from an ROI. Mitotic figures are in yellow circles.

Figure 2 shows examples of stromal contrast and nuclear pleomorphic contrast for two WSIs (Fig. 2c) with a low and a high BRACE risk score. Contrast measures the local variation in a feature where a low value represents less variations and a high value represents more variations. Higher stromal contrast (score = 20.81; Fig. 2d) and low pleomorphic contrast (score = 0.065; Fig. 2e) is associated with good prognosis (BRACE risk score = 0.249) where as low stromal contrast (score = 10.16) and high pleomorphic contrast (score = 12.58) is associated with bad prognosis (BRACE risk score = 10.38). Similarly, Fig. 3 shows digital tumour grade composition and mitotic count features. Higher percentage of LG3 (77%; Fig. 3b) and higher digital mitotic count (count = 60; Fig. 3c) are associated with poor prognosis (BRACE risk score = 10.38).

### DCIS filter and Region Segmentor

Tissue area was segmented using thresholding and morphological operations. DCIS filter (a trained CNN model) distinguished between invasive tumour regions and DCIS and achieved average F1 scores of 0.713 and 0.9 for invasive tumour and DCIS segmentation (Supplementary Table 3), respectively. Supplementary Fig. 2a shows an example of WSI-level tumour and DCIS segmentation output. Similarly, Region Segmentor (another trained CNN model) performed semantic segmentation of stromal and other non-ROI regions (fats, normal, blood vessels, artifacts) and achieved a dice score of 0.76 for stroma and 0.69 for other regions (Supplementary Table 3). The trained models were used to generate segmentation masks for the WSIs (Supplementary Fig. 2b).

### Tumour-rich area identification (Tumour Detector)

Tumour Detector (Supplementary Fig. 3; a cell segmentation and classification model) was used to generate WSI-level nuclei-contours (used to measure tumour-nuclei morphology) and types (to get tumour-nuclei density). The output of this module was then used for ROI selection for training the TGC module (Local Grade Predictor) and for counting mitotic figures. Supplementary Table 4 lists the three-folds cross-validation performance of different CNN models where Tumour Detector achieved the best F1 score of 0.79 in comparison to other models.

### AI-based prediction of grade composition (Local Grade Predictor)

Despite the fact that there is intra-tumour heterogeneity^2,40,41^, a single grade is often assigned to the entire BC case. Local Grade Predictor performed AI based grade prediction of individual patches within the WSI to capture variations due to intra-tumour heterogeneity. Tumour Detector was utilised to select an ROI based on tumour nuclei density, size and shape and instead of using patches from all over the WSI, patches from the selected ROIs were used to train Local Grade Predictor. Based on a linear support vector machine (SVM) trained with the proportions of digital local grades to predict the clinical grade of each WSI, the selection of ROI based on tumour nuclei density, size and shape improved receiver operating characteristic area under the curve (ROC-AUC) from 0.65 ± 0.023 (for baseline random ROIs) to 0.83 ± 0.014 (Supplementary Table 3). Another strategy where ROIs were selected from areas with maximum tumour tissue produced ROC-AUC of 0.79 ± 0.011. TGC considered local intra-tumour heterogeneity based on the proportion of areas in a given WSI that can be associated with grade 1–3. Supplementary Fig. 4 shows the association of the clinical grade and its components (mitotic score, tubule formation, and nuclear pleomorphism) with the TGC for both internal and external validation cohorts. It can also be observed that the Local Grade Predictor model does not only learn the overall grade but it also learnt the heterogeneity of clinical grade. LG1 predictions were present for all the three grades showing that at least some of the areas in most of the WSIs is similar to Grade 1.

### Digital mitotic count

A stain-robust mitotic detection model^42^ was used to detect mitotic figures. Mitotic figures were detected in an ROI from each WSI selected based on the same criteria as for training the Local Grade Predictor. The mitotic detection model first segmented potential mitotic figures and then refined the classification via a deep learning classifier. To assign a mitotic score to a WSI, an ROI from the WSI was passed through the model and the mitotic count was then used to decide the score. To reduce the effect of any under- or over-detections the counts were discretised to get a digital mitotic score from 1 to 3.

### Stromal and pleomorphic contrast

Stromal nuclei detection from Tumour Detector were used to construct a co-occurrence matrix (CM; Supplementary Fig. 5) where each entry represented the number of times a patch containing certain number of stromal nuclei co-occurred with another patch containing certain number of stromal nuclei. The CM was then used to calculate WSI-level contrast which quantified the local stromal variations. Another stromal nuclei related feature was also calculated which quantified the co-occurrences of patches with low stromal density. Similarly, the patch-level pleomorphic predictions from Local Grade Predictor were used to construct a CM containing entries for local pleomorphism which was then summarised at WSI-level as pleomorphic contrast.

### Survival analysis

The selected features i.e. TGC (LG1%, LG2%, LG3%), percentage of tumour area, pleomorphic contrast, stromal contrast, co-occurrences of stromal nuclei patches with low density, and digital mitotic score, were combined by Cox proportional hazard regression model to generate BRACE risk score. Supplementary Fig. 6 shows the contribution of each component of BRACE marker where higher percentages of LG1 and higher stromal contrast are associated with better outcomes whereas higher percentages of LG3, higher pleomorphic contrast, higher mitotic score and larger tumour area percentages are associated with worse outcomes. Table 1 shows the outcome results (*P* value of the log-rank test, Concordance or C-Index, and hazard ratio (HR) with 95% confidence interval (CI)) of the proposed BRACE marker and other clinical features on internal and external validation sets when used for stratifying patients into high-risk and low-risk groups in terms of DMFS and BCSS. In comparison to clinical grade, BRACE produced higher C-Indices (with significant *P* values of the log-rank test) for both DMFS and BCSS especially when generalising to the external cohort. Supplementary Table 5 shows the outcome results of BRACE on the discovery set. For comparison, Supplementary Table 6 also lists the outcome results of a larger set of features excluding the features included in BRACE. Similarly, to compare with a simple baseline DL model Supplementary Table 7 shows the outcome results of ResNet-18 (pretrained on ImageNet) used to extract features from the same set of patches as BRACE and a clear drop in performance was noted in comparison to BRACE.

**Table 1:** Results on internal and external validation sets.

Cohort-A (Validation set)				Cohort-B			
LN: Negative, Event: DMFS				LN: Negative, Event: DMFS			
**Feature**	***P*** **value**	**C-Index**	**HR (95% CI)**	**Feature**	***P*** **value**	**C-Index**	**HR (95% CI)**
Grade	0.0017	0.68 ± 0.06	1.83 (1.21–2.76)	Grade	0.0058	0.68 ± 0.07	2.16 (1.23–3.82)
NPI	0.0091	0.70 ± 0.06	2.00 (1.28–3.13)	NPI	0.0180	0.75 ± 0.06	2.72 (1.42–5.21)
BRACE	0.0010	0.73 ± 0.06	1.46 (1.22–1.74)	BRACE	0.0002	0.73 ± 0.06	1.22 (0.83–1.80)
**LN: 0–3, Event: DMFS**				**LN: 0–3, Event: DMFS**			
Grade	<0.0001	0.68 ± 0.04	1.97 (1.41–2.76)	Grade	0.0293	0.67 ± 0.05	1.93 (1.14–3.27)
NPI	0.0004	0.72 ± 0.05	1.45 (1.25–1.68)	NPI	0.0094	0.75 ± 0.06	1.77 (1.29–2.43)
BRACE	<0.0001	0.72 ± 0.05	1.49 (1.29–1.72)	BRACE	0.0003	0.68 ± 0.07	1.23 (0.86–1.76)
**LN: Negative, Event: BCSS**				**LN: Negative, Event: BCSS**			
Grade	<0.0001	0.74 ± 0.07	2.92 (1.72–4.96)	Grade	0.0023	0.69 ± 0.07	2.29 (1.27–4.12)
NPI	0.0001	0.82 ± 0.05	3.06 (1.98–4.75)	NPI	0.0069	0.76 ± 0.06	2.91 (1.49–5.68)
BRACE	<0.0001	0.84 ± 0.04	1.58 (1.34–1.86)	BRACE	0.0001	0.73 ± 0.06	1.24 (0.85–1.82)
**LN: 0–3, Event: BCSS**				**LN: 0–3, Event: BCSS**			
Grade	<0.0001	0.72 ± 0.05	2.61 (1.75–3.90)	Grade	0.0112	0.68 ± 0.06	2.06 (1.18–3.59)
NPI	<0.0001	0.80 ± 0.04	1.54 (1.32–1.80)	NPI	0.0085	0.74 ± 0.06	1.74 (1.24–2.44)
BRACE	<0.0001	0.79 ± 0.04	1.57 (1.37–1.81)	BRACE	<0.0001	0.73 ± 0.06	1.28 (0.91–1.80)

Internal validation set (Cohort-A), external validation set (Cohort-B). *P* value (of the log-rank test), C-index and hazard ratio (HR) with 95% confidence interval (CI) for the proposed BRACE marker and other clinical features for DMFS and BCSS on a subgroup of endocrine treated patients with LN− and LN 0–3 are listed. Events are censored at 10 years. *x* ± sd for the C-Index represents one standard deviation of the mean over 1000 bootstrap runs.

No clear effect of the scanner type on prediction accuracy was observed for the compared features (Supplementary Table 8). For example, for BCSS, both Grade and BRACE produced higher C-Indices on cases scanned with Pannoramic scanner as compared to Philips scanner whereas for NPI it was the other way around. For DMFS, C-Index of Grade was almost the same for both the scanners. Overall, NPI’s predictions were better for Philips scanner whereas both Grade and BRACE performed better for Pannoramic scanner. The effect of scanner type might become more obvious when studied in a larger cohort with sufficient number of events.

In another experiment two components of NPI i.e. lymph node status and invasive tumour size were included in BRACE (this experiment is denoted by BRACE* to differentiate from main BRACE). Except for DMFS (LN 0–3) of external validation set, BRACE* showed comparable or improved prediction performance for both DMFS and BCSS in internal as well as external validation sets (Supplementary Table 9).

**Fig. 4:**
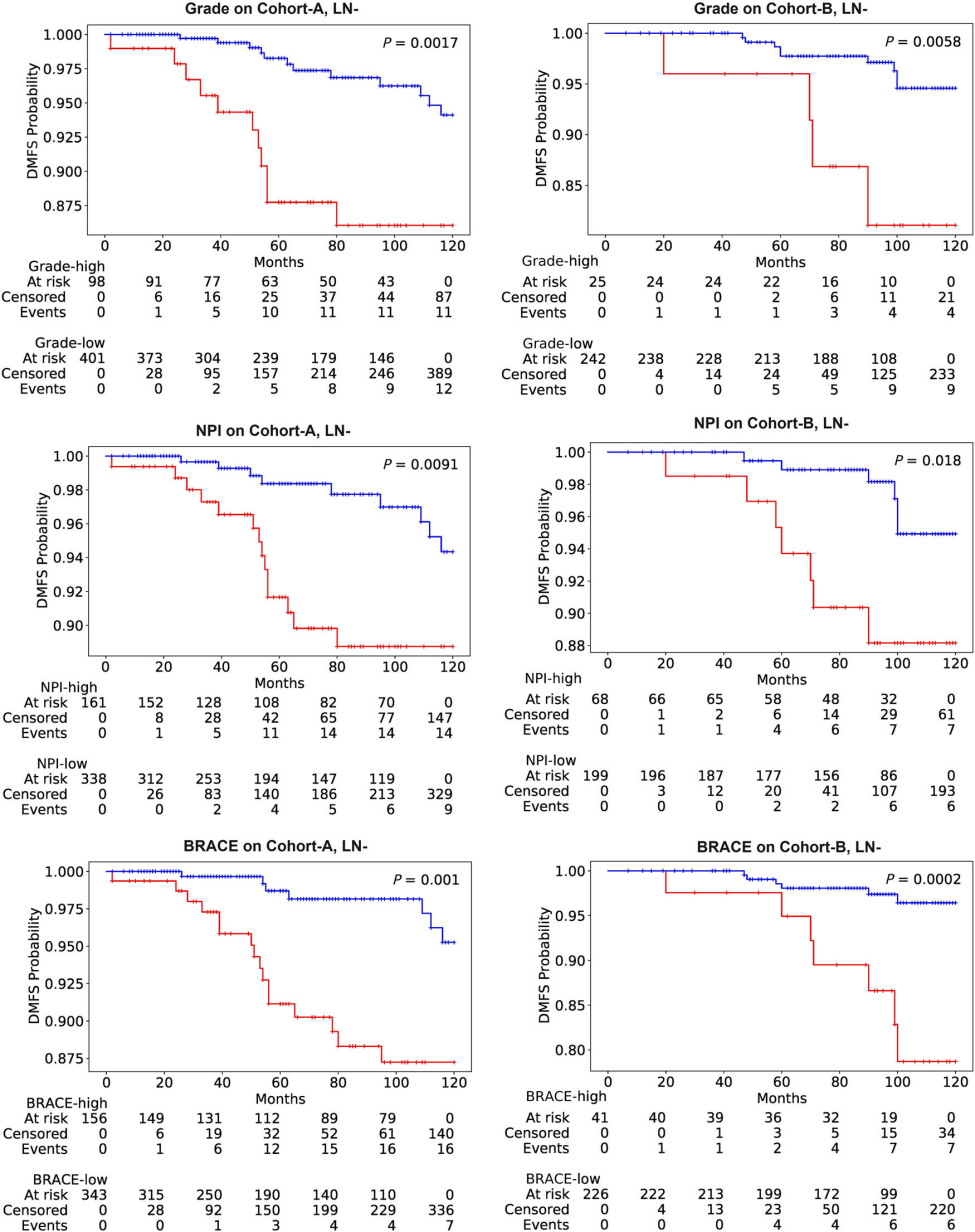
KM curves for LN− DMFS. KM curves for the high-risk (red line) and low-risk (blue line) groups of LN− DMFS as stratified by BRACE marker and other clinicopathological variables on the validation sets (Cohort-A: *n* = 499; Cohort-B: *n* = 267). *P* values are for the log-rank test.

Figure 4 shows Kaplan–Meier (KM) curves for DMFS in LN− cases using different clinicopathological features and BRACE marker on internal as well as external validation cohorts. The number of events in the high-risk group (*n* = 16) and low-risk group (*n* = 7) of BRACE were almost the same as in the NPI high- and low-risk groups but for clinical grade the proportion of the events was 11 (high-risk) to 12 (low-risk). This trend was more evident for the clinical grade on the external cohort where the number of events in the low-risk group were more (*n* = 9) than in high-risk group (*n* = 4) suggesting the limitation of a discrete grade risk score in stratification. Supplementary Fig. 7 and 8 shows KM curves for DMFS and BCSS, respectively, in the discovery set. Similarly, Supplementary Figs. 9–11 shows KM curves for DMFS (LN 0–3), BCSS (LN−), and BCSS (LN 0–3), respectively, in the validation sets.

### Identification of high-risk patients for additional chemotherapy

**Fig. 5:**
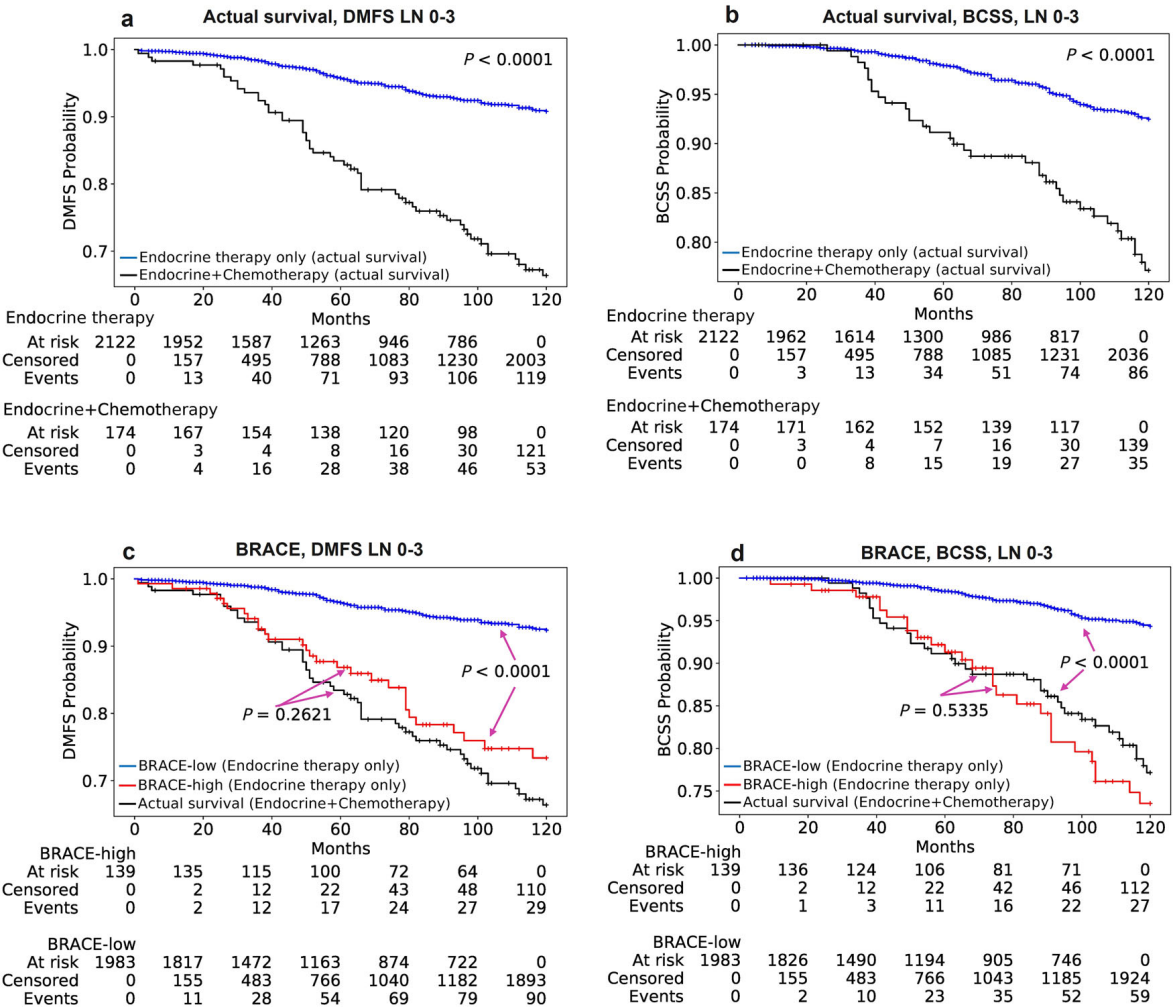
KM curves for identifying cases for chemotherapy (Cohort-A). KM curves for actual survival in endocrine therapy only treated (*n* = 2122) and endocrine+chemotherapy treated (*n* = 174) patients for endpoints DMFS (**a**) and BCSS (**b**). KM curves for high-risk (red line) and low-risk (blue line) groups of LN 0–3 as stratified by BRACE marker for endpoints DMFS (**c**) and BCSS (**d**) in patients treated with endocrine therapy only in discovery Cohort-A. With appropriate cut-off BRACE identified cases which could have benefited from additional chemotherapy as shown by the overlap of the predicted high-risk curve (red line) with the actual survival curve (black line) of cases treated with chemotherapy. BRACE-high and BRACE-low represents cases identified as high- and low-risk, respectively, by BRACE marker. *P* values are for the log-rank test.

A feature of BRACE marker is that it has shown the ability to predict high-risk patients who could benefit from additional chemotherapy. To test the predictive ability of BRACE marker, we compared the KM curves of high-risk group predicted by our model with the actual survival curve of patients who received additional chemotherapy. Figure 5a, b shows the actual survival of patients treated with endocrine therapy only and those treated with additional chemotherapy in the whole Cohort-A. Figure 5c, d show the overlap between the survival curves of predicted high-risk group (in red) in endocrine therapy treated patients with actual survival curves of high-risk chemotherapy treated patients (in black) with no significant difference (DMFS: *P* = 0.26, BCSS: *P* = 0.53) suggesting the ability of our marker to identify patients who can benefit from additional chemotherapy. Patients in both blue and red curves in Fig. 5c, d were treated only with endocrine therapy. Similarly, Supplementary Fig. 12 shows the same analysis but restricted to internal validation set.

### Multivariate analysis

**Fig. 6:**
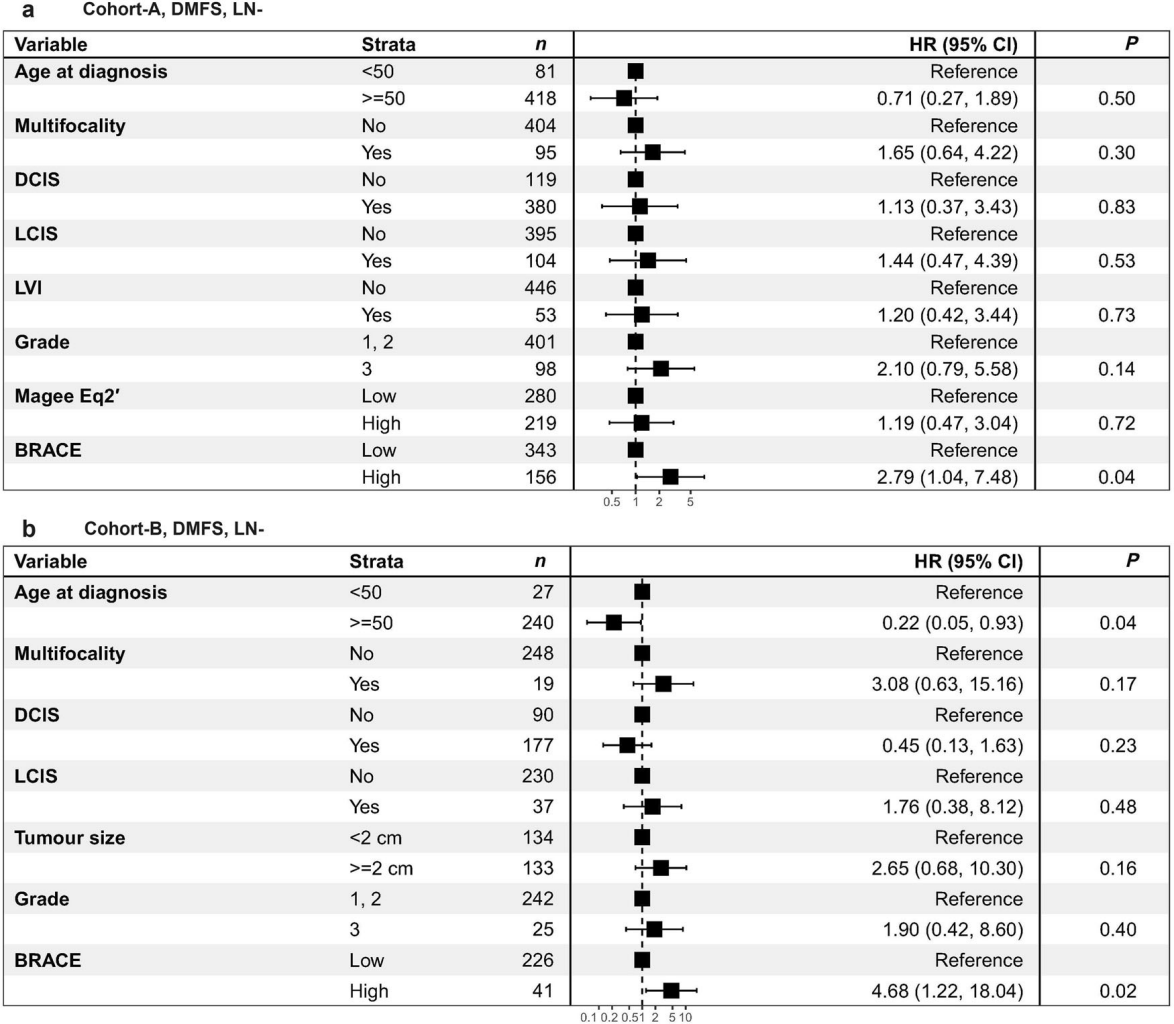
Multivariate analysis for DMFS, LN− cases. Forest plots showing the HR with 95% confidence intervals (CI) and *P* values (of the log-rank test) for BRACE marker when adjusted for other clinicopathological variables on DMFS (LN−) for internal validation set of Cohort-A (*n* = 499) (**a**) and external validation set Cohort-B (*n* = 260) (**b**).

The prognostic significance of BRACE marker was further investigated when adjusted for other clinicopathological variables in a multivariate analysis. Figure 6 shows the forest plots from multivariate Cox proportional hazard regression model for DMFS (LN−) cases where the HR along with their 95% confidence intervals are listed for both validation Cohort-A and Cohort-B. BRACE marker was found to be independent prognostic variable against other clinicopathological variables for DMFS in LN− cases of Cohort-A (*P* = 0.04, HR = 2.79, CI: 1.04–7.48; Fig. 6a) as well as Cohort-B (*P* = 0.02, HR = 4.68, CI: 1.22–18.04; Fig. 6b). Supplementary Fig. 13 shows Cohort-A forest plots for DMFS (LN 0–3) and BCSS (LN− and LN 0–3) where BRACE marker showed prognostic ability for DMFS (LN 0–3) cases (*P* = 0.006, HR = 3.09, CI: 1.39–6.87; Supplementary Fig. 13a), BCSS (LN−) cases (*P* = 0.018, HR = 6.39, CI: 1.37–29.83; Supplementary Fig. 13b) and BCSS (LN 0–3) cases (*P* = 0.010, HR = 3.95, CI: 1.40–11.16; Supplementary Fig. 13c). Grade was also found to be significant for BCSS (LN−) cases (*P* = 0.006, HR = 6.12, CI: 1.70–22.03; Supplementary Fig. 13b) and BCSS (LN 0–3) cases (*P* = 0.006, HR = 3.85, CI: 1.49–9.99; Supplementary Fig. 13c).

Similarly, Supplementary Fig. 14 shows forest plots for DMFS (LN 0–3) and BCSS (LN− and LN 0–3) on external Cohort-B. BRACE marker was found to be independent prognostic variable against other clinicopathological. For DMFS in LN 0–3 cases (*P* = 0.02, HR = 4.17, CI: 1.31–13.32; Supplementary Fig. 14a), BCSS in LN− cases (*P* = 0.02, HR = 5.38, CI: 1.32–22.00; Supplementary Fig. 14b), and BCSS in LN 0–3 cases (*P* = 0.008, HR = 5.52, CI: 1.58–19.32; Supplementary Fig. 14c). Grade was found to be not significantly associated with survival in the external validation. These results suggest that BRACE marker added information over and above other clinicopathological variable including grade.

### Comparison with Magee equation

The predictive ability of BRACE marker was indirectly compared in terms of C-index with ODX risk score via the results of a previously published study where *New Magee equation 2*^43^ showed a moderate correlation (Pearson’s correlation coefficient r = 0.604) with ODX risk score. This equation is based on clinicopathological variables including NPI, tumour size, ER HScore, PR HScore and HER2 status. For Cohort-A PR HScore was missing for Magee equation. On the validation set of Cohort-A, in comparison to BRACE marker’s C-indexes of 0.73 ± 0.06 and 0.84 ± 0.04 for LN− DMFS and BCSS, respectively, Magee equation produced C-indexes of 0.69 ± 0.05 and 0.78 ± 0.04 suggesting better risk ranking by the former.

### Correlation with clinicopathological parameters

BRACE marker was significantly associated larger tumour size (*P* < 0.0001), high tumour grade (*P* < 0.0001) including high pleomorphism (*P* < 0.0001), low tubule formation (*P* < 0.0001), and high mitotic scores (*P* < 0.0001). It also showed significant association with high NPI score (*P* < 0.0001; Supplementary Table 10). It was significantly associated with lymph vascular invasion (LVI) on Cohort-A but the association was not significant on Cohort-B. No associated was observed for different age groups, menopausal status and different BC types.

## Discussion

The decision of chemotherapy administration to patients with early-stage ER+/HER2− BC is critical to avoid unwarranted chemotherapy side effects. The current methods in histopathological analysis are mostly subjective or based on gene expression profiling which is expensive and time consuming. In this study, we developed an AI-based method (BRACE marker) which can identify patients in need for chemotherapy in this intermediate risk group using an objective and reproducible method reducing the effect of variations present in manual clinical grading. Furthermore, BRACE marker utilises H&E slides used in routine clinical practice, so no extra sampling is needed saving labour and costs of gene expression assays.

By using AI and image analysis, BRACE pipeline extracted a rich set of features including tumour morphology, tumour–stroma relationship, TILs quantification, phenotypic heterogeneity and mitotic activity from H&E-stained breast cancer images. These important biologically driven features cannot be objectively quantified in accurate manner by current BC grading. Capturing more detailed morphological patterns in the form of grade proportions and the features based on such local grades when combined with tumour area percentage, and pleomorphic and stromal variations can help predicting DMFS and BCSS. BRACE followed a bottom-up approach where pathologists’ supervision in the form cellular and regional annotations was utilised so that the results are explainable as well as appropriate for the prognostication task. For example, the method can quantify how much pleomorphic or stromal variation is present in a WSI or what are the proportions of different grades along with their locality.

Our results on univariate analysis (Table 1) showed that the proposed BRACE marker can rank (in terms of C-index) patients at risk of distant metastasis as well as BC-specific death better than conventional manual clinical grade and comparable to NPI. More importantly these results suggested better generalisation of BRACE marker to external cohort as compared to clinical grade. From the multivariate analysis it was observed that BRACE marker adds clinically relevant information over other clinicopathological variables including tumour size, age at diagnosis, and grade. Although, the Local Grade Predictor in our proposed pipeline was trained in a supervised way by utilising the clinical grade as WSI-level label, our BRACE marker ranked patients better in terms of C-Indices in comparison to the clinical grade for the following main reasons: (a) by training the local grade composition model only on ROIs selected by a criteria of high tumour nuclei density and high tumour nuclei eccentricity; (b) by capturing more detailed morphological patterns in the form of local grades (grade proportions); (c) by incorporating tumour region proportion, and pleomorphic and stromal variation.

Both inter- and intra-tumour heterogeneity have effects on patients outcome and response to therapy^2,3^. These variations include tumour differentiation, cellularity, stromal and immune response, tumour architecture and histological tumour type. Such heterogeneities pose challenges for quantification and hence obtaining a single risk score for a case is not possible from visual assessment. It has also been shown that high heterogeneity of tumours is associated poor prognosis because of less immune cell infiltration^44^. With the power of AI a detailed information can be obtained in an objective manner and multiple features can be integrated to a single score which can then be used for prognosis. BRACE pipeline extracted a rich set of features from WSI which can help in a plethora of further research explorations. BRACE quantified the overall nuclear heterogeneity with in a WSI into one score (Pleomorphic Contrast) where a higher score, i.e. more local pleomorphic variations, was associated with poor prognosis as also reported in previous studies^2,3,44^.

Stroma-to-tumour ratio (STR) has been shown to have independent prognostic relevance in different tumours including BC^45,46^. However, the relevance of STR still needs further research in BC because some studies have shown association of high STR with poor prognosis^45^ while other demonstrated good prognosis^46,47^. These inconsistencies might be attributed to the variations and subjectivity in the assessment of tumour stroma as well as the heterogeneous nature of BC. BRACE feature set included a quantitative assessment of stromal cell ecology and entropy in terms of Stromal Contrast measuring the local variations in stromal cells in a WSI and may provide an alternative for STR quantification. High stromal cell variations was associated with good prognosis and vice versa.

Another DL-based histological grading work (DeepGrade^32^) related to BC survival analysis that was recently published is in line with BRACE but differs from this work in the following aspects. DeepGrade stratified only Grade 2 patients whereas BRACE produced a composition of all grades along with other image-based features. The main outcome for DeepGrade was recurrence-free survival but for BRACE both DMFS and BCSS were the main outcomes. BRACE followed a bottom-up approach (region segmentation, cell segmentation and classification, followed by features generation) for producing interpretable features as compared to the only deep features of DeepGrade. Furthermore, DeepGrade utilised an ensemble of twenty CNN models for grade prediction whereas one CNN model was used for local grade prediction in BRACE.

To achieve better stratification of patients, BRACE combined multiple important histological features. This idea was supported by a previous study^35^ analysing the correlation between ODX score and DL based mitotic count. Using frequency of mitotic figures, a linear SVM classified a patient as either a high- or low-risk. Their analysis showed that mitotic count cannot be used alone for risk stratification of intermediate risk group suggesting addition of other pathologic features. The digital mitotic count of BRACE was higher for high BRACE score than low BRACE score (Supplementary Fig. 15) and a higher mitotic score corresponded to higher risk which was again supported by the lower mean number of mitoses in low ODX vs higher mean number of mitoses in high ODX in their study^35^. The importance of mitotic figures for predicting ODX was also shown in another study^36^.

For explainable results BRACE followed a bottom-up approach where region and cell level information was used to generate high level features in different categories. A similar approach was adopted in another work^36^ where DL based features were generated in three main categories related to structures, cell types, and tissue types.

The ROI selection for training the local grade model in BRACE was based on tumour cell density and eccentricity to ensure the model learnt features representative of tumour patterns. A similar approach of ROI selection was adopted in another work^37^ where the ROIs for counting tumour and immune cells were based on high tumour cell density. Their study also corroborated the benefit of the usage of DL features in BRACE by reporting an improvement in correlation with ODX score by adding DL feature to Magee features. To generate interpretable model, feature selection in this study^37^ was mainly based on domain knowledge. Similarly, another work^38^ weighted the recurrence score predicted from H&E image tiles by tile-level tumour likelihood and combined with clinicopathological characteristics.

Unlike BRACE most of these studies used ODX score as a label for training a regression model and combined DL features with other clinicopathological parameters. A more related work^39^ in a similar line as BRACE used DL to extract features related to the three components of NGS and demonstrated it prognostic significance.

Limitations of this study included its retrospective nature because of the challenges associated with designing and conducting prospective studies. However, the method was validated on a large cohort of more than 2100 cases and its generalisability was also validated on an external cohort. With the availability of more data, a multicentric training and validation will be more useful for developing a robust model. Tumour and stromal architectures which could potentially add to a more significant indicator were not included in this work. Furthermore, due to unavailability of multigene assays, the method was indirectly compared with ODX via previously published results of Magee equation in terms of ranking the patients with C-index.

In conclusion, BRACE marker is an AI-based method which can identify high-risk patients in the intermediate risk group of ER+/HER2− with high significance, adds clinically relevant information over routine manual histological features, and provides a potential reproducible and cost-effective alternative to existing gene-based methods. This work should encourage further research in image-based prognostics in BC and other types of cancer. Our future plan is to investigate other image-based features such as the arrangements of tumour cells and stromal structures and apply the proposed method to other types of cancers (such as prostate). With access to multigene assays the method could further be validated for predictiveness and even a prospective study could also be designed for validation. H&E image-based features could also be combined with features from other stained images such as IHC as well as other clinicopathological features.

## Methods

### Datasets

This study included a large well-characterised luminal (ER+/HER−) BC cohort (*n* = 2122) who had received endocrine treatment, without chemotherapy, collected from the Nottingham University Hospital, Nottingham, UK from 1998–2020. This cohort (called Cohort-A) was used for discovery and internal validation. To validate the generalisability of BRACE marker, an external validation cohort (*n* = 311), referred here as Cohort-B, with same BC subgroup and clinicopathological data as Cohort-A was collected from University Hospital Coventry and Warwickshire (UHCW), Coventry, UK from 2011 to 2014. Distant metastasis-free survival (DMFS), i.e. time from surgery to development of the distant metastasis, and BC specific survival (BCSS), i.e. time from initial diagnosis to the time of BC related death, were the two endpoints of the analysis. Median follow-up duration for Cohort-A for DMFS and BCSS was 80 and 83 months, respectively, whereas for Cohort-B it was 96 months for both DMFS and BCSS.

Clinicopathological data (Supplementary Table 1) of female patients with age at diagnosis varying from 20–92 years included: lymph node (LN) status, clinical histological grade, tumour size, lympho-vascular invasion (LVI), Nottingham Prognostic Index (NPI), progesterone receptor (PR) status, follow-up and treatment data. Representative sections of formalin fixed paraffin embedded tissue blocks of surgical excision specimens from each case were H&E stained and scanned with Philips UFS scanner with 0.25 µm/pixels at ×40 to produce WSIs (*n* = 1417). A subset of cases (*n* = 705) were scanned using Pannoramic 250 Flash III; 3DHistech, Budapest, Hungary. For each patient one H&E-stained WSI was utilised for developing an AI-based BRACE marker for survival prediction.

Cohort-A was divided into discovery (*n* = 1496) and internal validation (*n* = 626) sets (Supplementary Fig. 1a). Three different splits were formed from the discovery set for cross-validation (Supplementary Fig. 1b). Supplementary Fig. 1b shows the detail of Cohort-B. A subset of cases (*n* = 174) from the source hospital of Cohort-A who received both endocrine and chemotherapy was used as a control group. To keep the evaluation fully blinded the survival times and events of validation sets were hosted on a webserver.

### Ethics statement

This study was approved by the Yorkshire & The Humber - Leeds East Research Ethics Committee (REC Reference: 19/YH/0293) under the IRAS Project ID: 266925. Data collected were fully anonymised.

### WSI annotations for various morphological features

To develop DL models for nuclei detection and invasive tumour/stroma/DCIS regions segmentation annotations were marked by six experienced pathologists for different regions and nuclei types^48^. Annotations were marked both at nuclei-level as well as region-level. The main nuclei type annotations included tumour, normal epithelial, stromal, and immune nuclei. Region annotations included tumour, tumour associated stroma (TAS), DCIS and lymphoid stroma etc. Annotations included in this study were about 756 mm^2^ of tumour area, 395 mm^2^ of DCIS area, 360 mm^2^ of stromal area, and 123,924 tumour nuclei. These annotations were used for training and evaluating the CNN models (DCIS filter, Tumour Detector, Region Segmentor). For training Local Grade Predictor the original slide-level clinical grade was used as a label. The proportion of clinical grades were grade 1 (23%), grade 2 (57%), and grade 3 (20%).

Due to availability of annotations for selected set of cases and the amount of data needed to train the different upstream modules of the proposed pipeline, different subsets of Cohort-A were used in training and validation (Supplementary Table 3). However, it was made sure that the feature discovery was done only on the discovery set. The trained models were applied to the whole slide for generating different features.

### DCIS filtering

The WSI at a low resolution was converted to HED (Haematoxylin–Eosin–DAB) colour space and entropy was calculated for each colour channel using rank filter. The diaminobenzidine (DAB) channel entropy was then subtracted from the sum of H and E channels and Otsu thresholding was applied to the resulting entropy. Different morphological operations (dilation, erosion, hole filling)^49^ were performed to get the desired tissue area. In order to exclude DCIS areas from the downstream analysis an EfficientUnet-based semantic segmentation model (DCIS filter) trained on pathologist annotated tumour and DCIS areas was applied to the tissue area (Supplementary Fig. 2a). Predicted DCIS areas were excluded from all the other downstream steps. All the models were trained and validated only on the discovery sets and the validation sets were only used for final validation.

### Tissue region segmentation

To segment stromal and other tissue regions the Region Segmentor was trained on patches from pathologist annotated areas of tumour, stroma, fats, etc. and tissue masks were generated for WSIs (Supplementary Fig. 2b). Note here that a separate model was employed for the region segmentation so that DCIS filter could concentrate more on accurate segmentation of tumour and DCIS only instead of also including stromal and other tissue regions (Supplementary Fig. 2c). As Region Segmentor segmented out stromal regions irrespective of them being associated with tumour, therefore to restrict to TAS only the following four steps were taken: (1) Tumour regions segmented by DCIC filter were combined with stromal regions segmented by Region Segmentor; (2) for fast processing the mask size was reduced by a factor of seven; (3) the tumour regions were dilated to capture the TAS with a disc of radius 8 pixels followed by filling the holes with a disc of radius 32 pixels; (4) any stroma captured in the dilated tumour regions was considered as TAS for further features calculation. Training parameters for Region Segmentor were as follows: patch size: 512 × 512 with 96 pixels context on all sides; batch size 8; learning rate 0.01 (initial five epochs), 0.001 (epoch 6–10), 0.0001 (epoch 11–30); momentum 0.9; cross entropy loss. Other settings: input was normalised to [0,1] and different augmentations (random brightness/contrast, random rotate, median blur) were used during training with values of 0.5.

### Tumour-rich area identification

To segment and classify different types of nuclei a state-of-the-art HoVer-Net^50^ (an inference version also available at https://github.com/simongraham/hovernet_inference) pretrained on the BC subset of the PanNuke^51^ dataset was fine-tuned on the target dataset resulting in Tumour Detector. As the nuclei-level annotations on WSIs marked by the pathologists were in the form of points, therefore, the segmentation masks generated by the pretrained HoVer-Net were used in combination with the nuclei types to further fine-tune the model on the target images. Input patches (*n* = 3200) of size 256 × 256 pixels from the target domain (discovery set of Cohort-A) were used for fine-tuning Hover-Net (Supplementary Fig. 3a). An additional patches (*n* = 400) from normal WSIs were used to augment the normal epithelial class. For the first 2 epochs only the decoders were fine-tuned with a learning rate of 0.0001 and for the subsequent 28 epochs both the decoders and the encoder were fine-tuned with a learning rate of 0.00001. Based on threefold cross-validation performance listed in Supplementary Table 4 Tumour Detector (i.e. the fine-tuned Hover-Net) produced best F1 of 0.79 and this was used for nuclear detection and classification. The trained model was then used to generate WSI-level nuclei-contours and types. The output of this module was used for ROI selection for training the Local Grade Predictor and for mitosis detection.

### Digital local tumour grade prediction

To train a DL Local Grade Predictor (Inception V3^52^ enhanced by adding two linear activation layers with ReLU activation and a fully connected layer) for predicting TGC for a WSI, clinical grades at WSI-level were available as labels/ground truth. But as diverse regions (such as stroma, fats, etc.) were also present in a WSI therefore training the model with a WSI-level label applied to all the areas did not perform well. To reduce the heterogeneity of region-level labels (i.e. a single WSI-level label assigned to all regions) so that the model is trained on the most relevant areas from the tissue, an automated approach for ROI selection was employed. Tumour Detector was utilised to select an ROI based on tumour nuclei density, size and shape and instead of using patches from all over the WSI, patches from the selected ROIs were used to train Local Grade Predictor. Each ROI was of size 5600 × 5600 pixels (about 1344 µm) at 40× magnification. Patches of size 512 × 512 pixels (about 123 µm) at 40× magnification from the ROIs were used for training Local Grade Predictor to predict TGC i.e. grade for an entire WSI at the patch-level.

### ROI selection

Based on three folds cross-validation, different strategies were evaluated for ROI selection including random ROI, ROI with maximum tumour tissue, and ROI with high tumour nuclei density and eccentricity and the last strategy was adopted because of its highest ROC-AUC for predicting clinical grade for a WSI. This empirical ROI selection criterion (eq1) gave more weightage to areas with larger and more deformed tumour nuclei.

1 $$\begin{array}{l}{{ROI}}_{{score}}={Tumour}\,{nuclei}\,{count}+2\left(\bar{x}\,{Tumour}\,{nuclei}\,{area}\right.\\ \left.\qquad\qquad\times\,\bar{x}\,{Tumour}\,{nuclei}\,{eccentricity}\right)\end{array}$$

where $\bar{x}$ represents the mean of tumour nuclei (area or eccentricity). Eccentricity measures how much a nuclei deviates from being circular. An ROI of size 5600 × 5600 pixels (about 1344 µm) at ×40 magnification with the highest ROI_score_ was selected from each WSI for training DL-based Local Grade Predictor. While sliding an ROI-sized window over the WSI for selecting the high score ROI an overlap of 50% was added.

### Patch selection

The extracted ROIs were cut into smaller patches of size 512 × 512 pixels (about 123 µm) at ×40 magnification so they can fit into computer memory to train Local Grade Predictor. To allow the model to pay more attention to tumour morphology, patches below a threshold of fifteen tumour nuclei were discarded. The threshold was selected based on the discovery set of each of the three splits.

### Local Grade Predictor training

To train a model for predicting TGC, we enhanced the performance of Inception V3^52^ by adding two linear activation layers with ReLU activation and a fully connected layer for predicting the grade and pleomorphism for an input patch (termed as Local Grade Predictor). The model used ImageNet^53^ pretrained weights for the unmodified layers. One ROI per WSI from the discovery set of each split was used for training and the validation set was used for model selection. Training parameters for Local Grade Predictor were as follows: batch size 8; learning rate 0.001; momentum 0.9; cross entropy loss. Other settings: input was normalised using ImageNet mean and standard deviation and different torchvision library’s augmentations were used during training: random crop (default parameters), random horizontal flip (*P*: 0.5), and colour jitter (brightness: 0.3, contrast: 0.3, saturation: 0.3, hue: 0.3). The trained model was then used to predict TGC for an entire WSI at the patch-level.

### Stromal and pleomorphic contrast

Spatial co-occurrence quantified the co-occurrence of two or more structures (such as tumour cells with stromal cells) within a certain distance in a patch of size 256 × 256 pixels. Once co-occurrence matrices (CM) were constructed, different features were calculated using the python library (skimage.feature.greycoprops-Scikit-image). Two of the main features based on CM included stromal cell contrast and pleomorphic contrast where contrast measures local changes of a feature over the WSI. It ranges from 0 (a constant image) to (size of CM—1). To calculate stromal contrast the co-occurrence of each stromal cell, in each patch of size 256 × 256 pixels, with any other cell at eight different angles were counted and put as entries in CM. A standard python library (greycoprops) was used to calculate different properties (contrast, dissimilarity, homogeneity, etc.) which served as a compact summary of the CM for each WSI. Pleomorphic contrast was calculated in a similar manner where the patch predictions in the form of local pleomorphic 1 (LP1), LP2 and LP3 from Local Grade Predictor were used to count the co-occurrence of these predictions. Supplementary Fig. 5 further explains how CM was constructed.

### Feature selection

A set of features (*n* = 700) in different categories (Supplementary Table 2) was extracted to identify features which could be explained from clinical point of view and perhaps could also be applied to other subgroups of BC. The prognostic importance of the features for ER+/HER2− patients was assessed by Cox L1 regression in terms of C-Index, *P* value (of the log-rank test) and HR on the discovery set and eight features (i.e. percentages of digital local grade LG1%, LG2%, LG3%, percentage of tumour area, pleomorphic contrast, stromal contrast, co-occurrences of stromal nuclei patches with low density, and digital mitotic score) were selected for final model development.

AI based grade has proven to be a prognostic marker for BC survival prediction by identifying useful morphological patterns^32,54^. To quantify grade at a detailed level and to put it in relation with overall tumour area, BRACE included the percentages of local grades and overall tumour percentage. Although nuclear pleomorphism is an important component of BC grade but due to high inter-observer variability^16,55^, it needs better quantification. To subjectively quantify the overall nuclear heterogeneity with in a WSI into a single score, BRACE included pleomorphic contrast which measures the local variation in nuclear pleomorphism where a low value represents less variations and a high value represents more variations. Similarly, mitotic count has been a well-known prognostic marker^35,56,57^ therefore its digital counterpart has been included in BRACE. Recently, the importance of stromal variations has also been found to be of prognostic significance^45,46,58–60^. To represent a quantitative measure of stromal cell ecology and entropy BRACE included stromal contrast and co-occurrences of stromal nuclei patches with low density, respectively.

It was noted that adding clinically relevant features from Category C (related to TIL features) and other ML features from Category F (different types of cell counts) did not add further information. The former might be attributed to the unestablished utility of TILs for the subgroup of ER+/HER2−, whereas the latter would be less useful because of the difficulty of reducing high variant cell counts to a single value at WSI-level.

### Statistical analyses

To identify the prognostic ability of the proposed BRACE marker, a Cox proportional hazard regression model (from lifelines package for python - https://lifelines.readthedocs.io/en/latest/fitters/regression/CoxPHFitter.html) was fitted on three different splits of the discovery set. After the feature and parameter selection a single model was fitted on the whole discovery set. Parameters for the model were set as: estimation method (Breslow), L1 (0.5), L2 (0.5), and penalty (0.001). The fitted model was then evaluated on the internal and independent external validation cohorts. The regression coefficients were used to compute predictive risk score for each patient. KM curves were used to show risk stratification and a *P* value < 0.05 for two-tailed log-rank test was considered as significant. Based on three splits discovery sets the cut off for BRACE maker was set at 70th percentile. Similarly, for clinical variables such as grade, grade 1–2 was taken as reference against grade 3, whereas the cut off point for NPI was set at 60th percentile. Forest plots were generated using R function ‘coxph’ and R-package ‘forestmodel’. Chi-square test was used for analysis of categorical data.

### Reporting summary

Further information on research design is available in the Nature Research Reporting Summary linked to this article.

### Supplementary information

Supplementary material

REPORTING SUMMARY

## Data Availability

Histology image data and associated clinical metadata for the discovery and internal cross-validation cohorts can be obtained through PathLAKE data access request [https://www.pathlake.org/pathlake-data/].

## Code availability

The code for reproducing the results for external cohort is available at https://github.com/TissueImageAnalytics/BRACE-marker.
