# Supplementary material for: AI-enabled routine H&E image based prognostic marker for early-stage luminal breast cancer
Source: NPJ Precis Oncol. 2023 Nov 15;7:122. doi: 10.1038/s41698-023-00472-y (PMC10651910; doi:10.1038/s41698-023-00472-y)
Supplement: Supplementary file 1 — Supplementary material [file 41698_2023_472_MOESM1_ESM.docx]

**AI-enabled routine H&E image based prognostic marker for early-stage luminal breast cancer**

Noorul Wahab *et al*

**Supplementary Material**


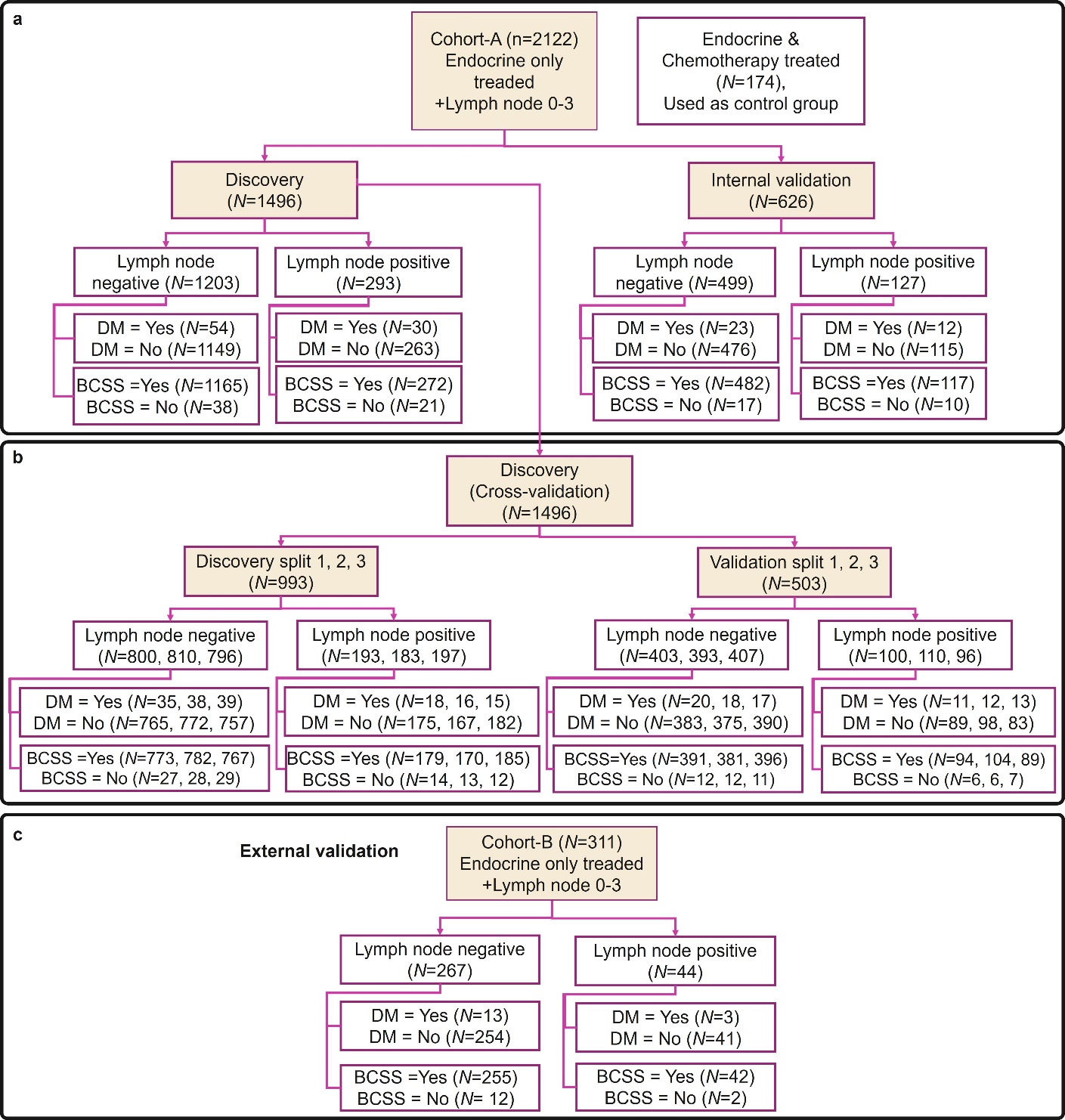


**Supplementary Figure 1 Discovery and validation sets.** (a) Division of Cohort-A into discovery and internal validation sets. (b) Further splitting of Cohort-A discovery set into three splits for cross-validation. (c) Cohort-B for external validation. Number of events (DM=distant metastasis, and BCSS=breast cancer specific survival) are at 10 years of censoring.


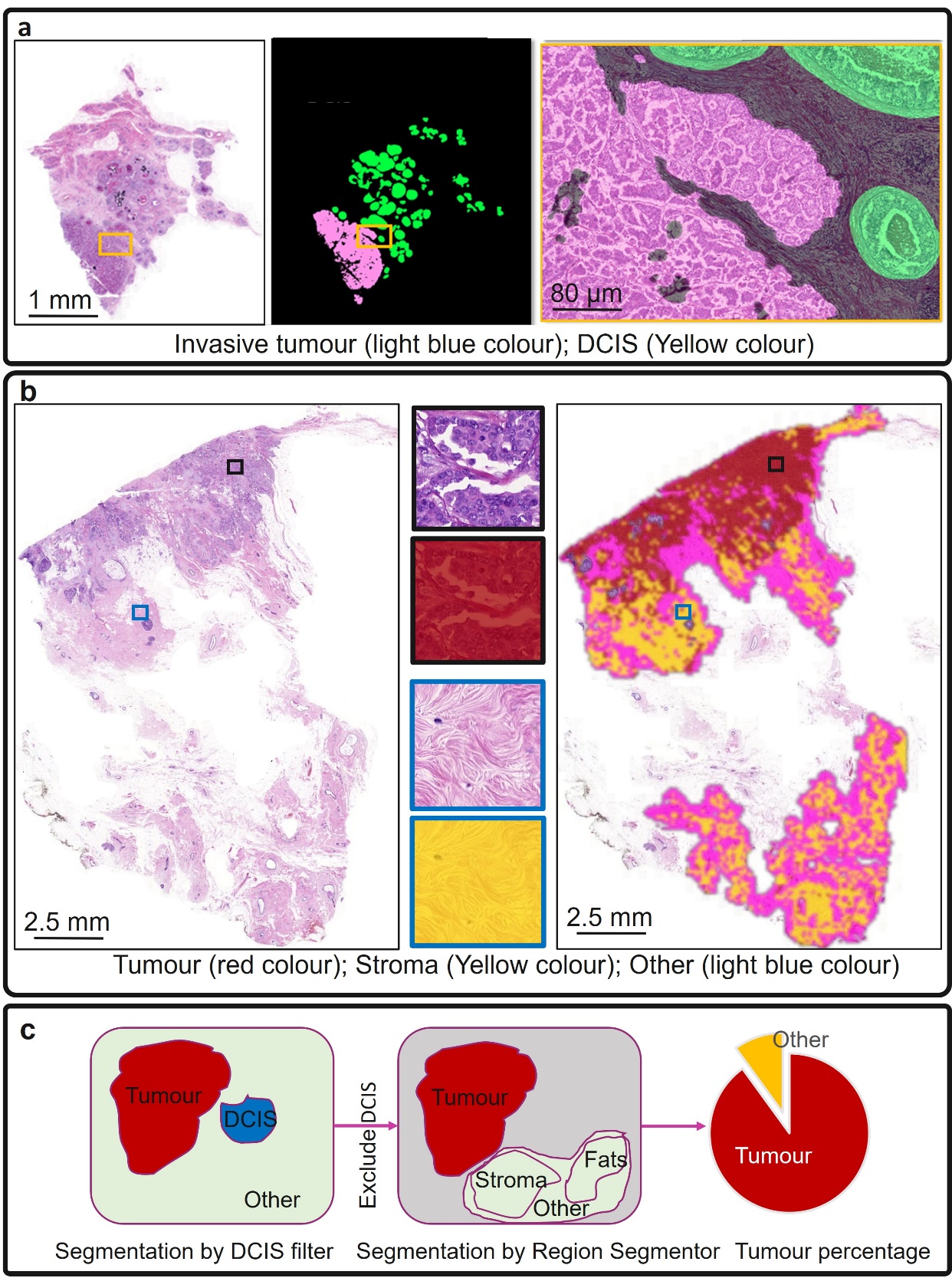


**Supplementary Figure 2 Tissue region segmentation (**a) Semantic segmentation of invasive tumour and DCIS regions by DCIS filter; (b) Tissue regions were classified as tumour (from DCIS filter), stroma, and other by Region Segmentor and the proportion of tumour region was then used along with other features of BRACE marker; (c) For accurate semantic segmentation of DCIS regions DCIS filter was restricted only to tumour and DCIS regions. Region Segmentor then classified the tissue area, excluding DCIS, into different regions to estimate tumour proportion.


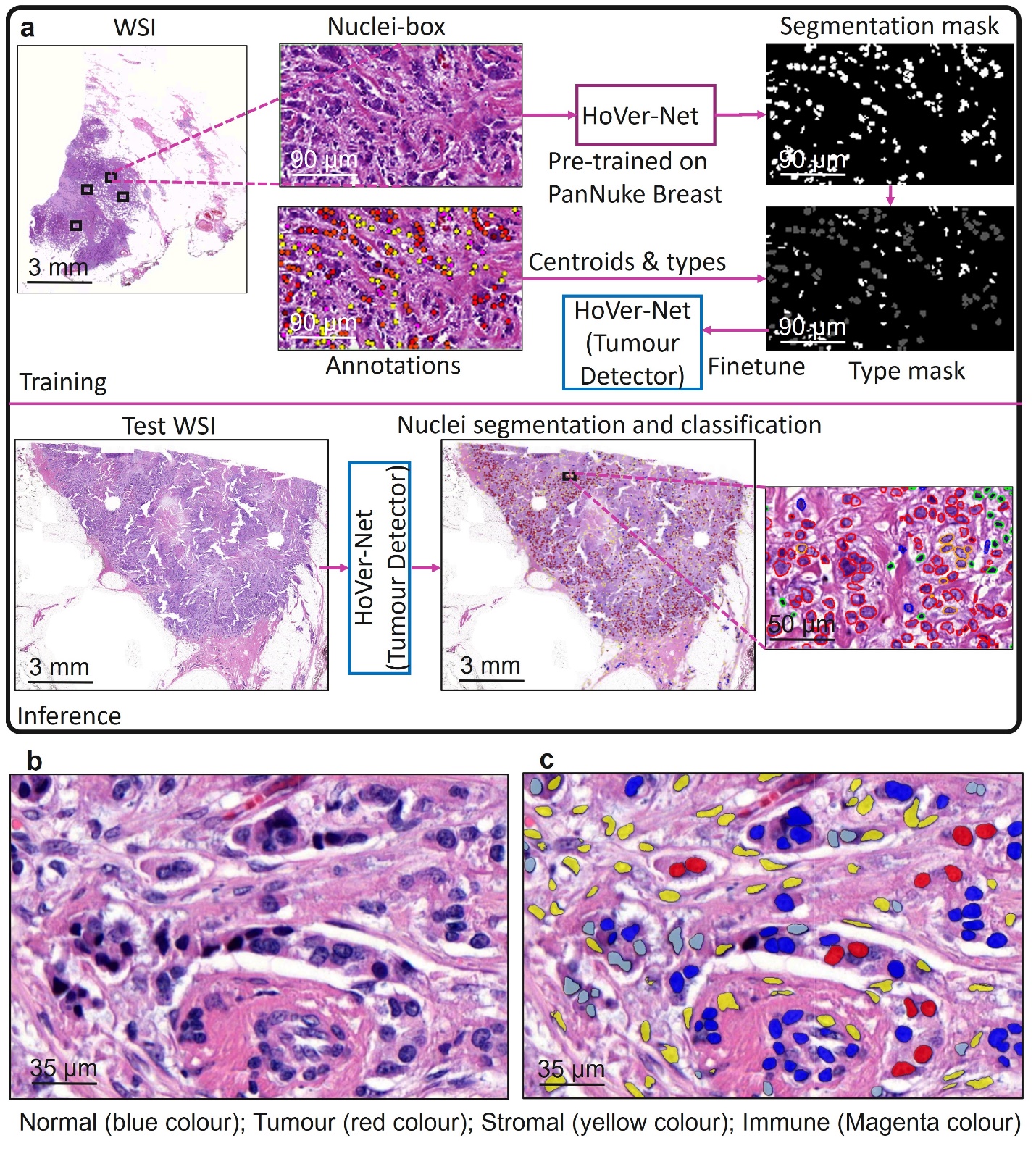


**Supplementary Figure 3 Fine-tuning of HoVer-Net for cell segmentation and classification to get Tumour Detector.** (a) Nuclei-box: is an area where a pathologist annotated different types of cells in the form of dots; Type mask: pixel-wise classes (types) of different cells segmented by the pre-trained model. (b) A sample patch. (c) Qualitative results of cell segmentation and classification on patch in (B).


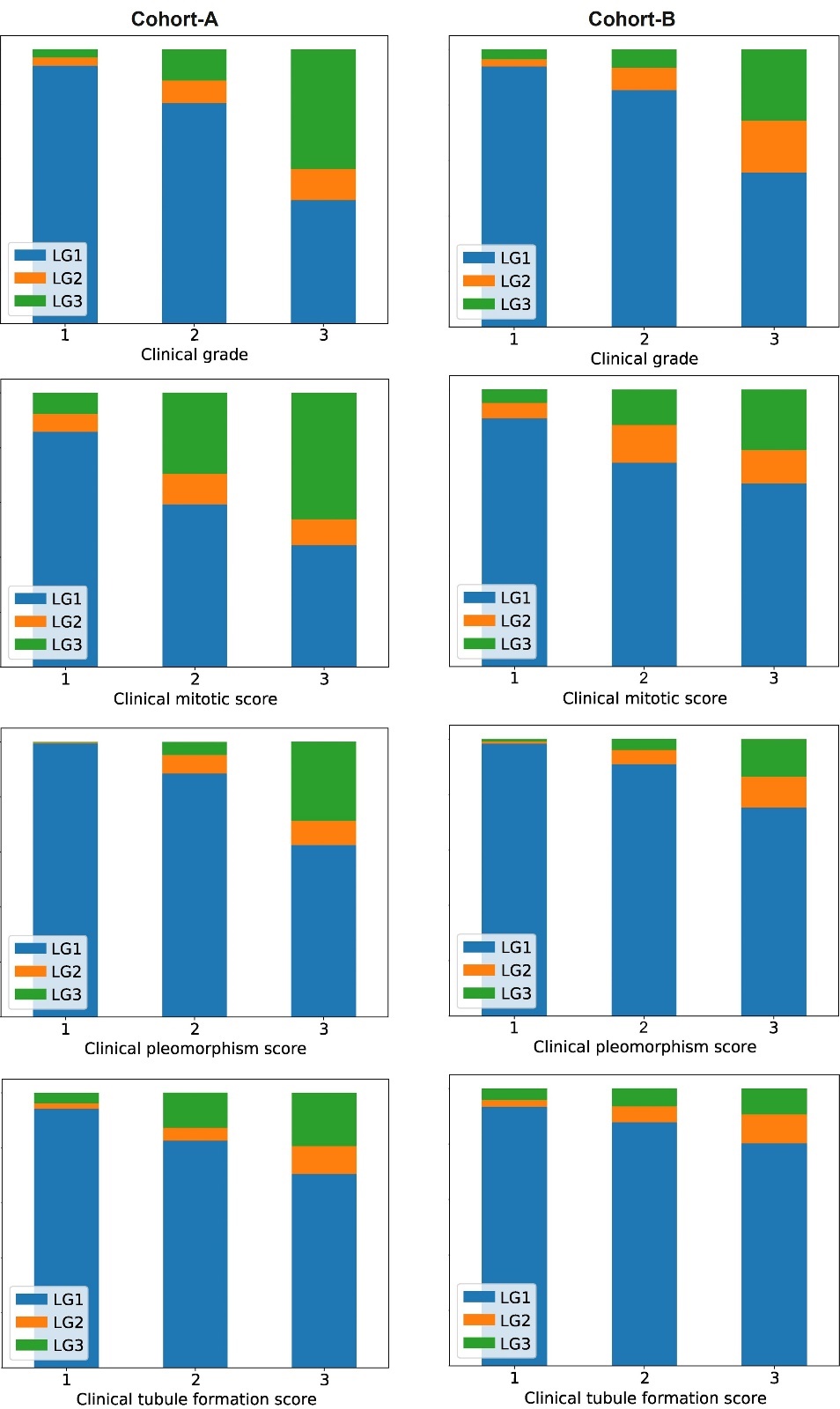


**Supplementary Figure 4 Concordance between TGC and clinical grade and its components:** The bar charts show that for majority of WSIs a high proportion of TGC corresponds to their respective clinical grade and its components. Low score (1), intermediate score (2), and high score (3).


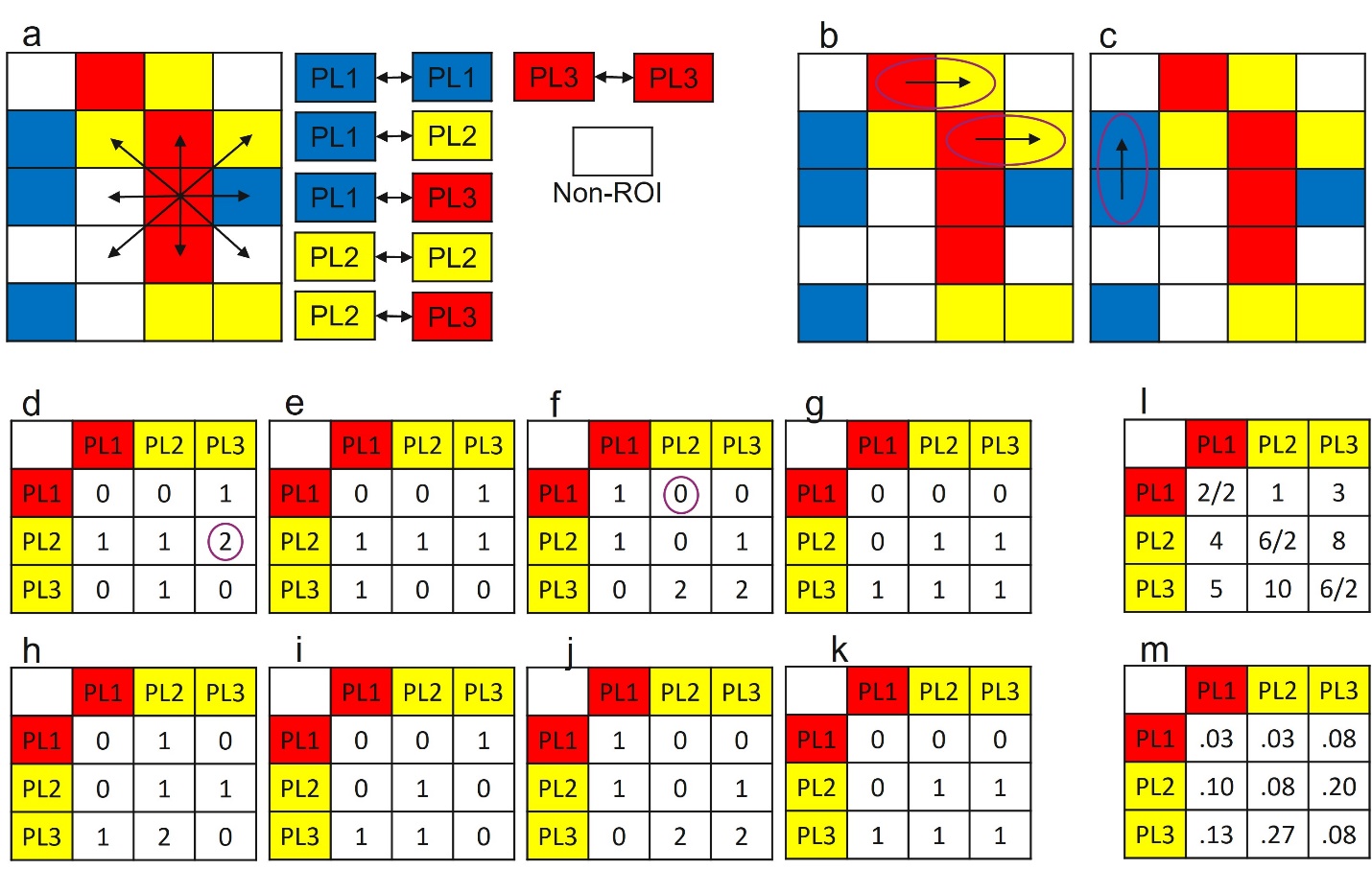


**Supplementary Figure 5** Construction of co-occurrence matrix (CM). (a) Possible co-occurrences of patches predicted as pleomorphic 1 (PL1), PL2 or PL3. Occurrences of PL2 to the right (i.e., at angle 0) of PL3 are 2 as shown by ellipses in (b) and the count is circled in (d). Similarly, PL1 occurring above (i.e., at angle 90) PL1 is 1 and is shown by an ellipse in (c) and the count is circled in (f). Such occurrences can be calculated at 8 different angles 0, 45, 90, 135, 180, 225, 270, and 315 shown from (d) to (k) and are summed to get total co-occurrences (l). Note: to get the sum of occurrence of the same entity for example PL1 with PL1, the total co-occurrences are divided by 2. The resulting values are then normalized and the resulting CM (m) is then used to calculate different features such as homogeneity), contrast, etc.


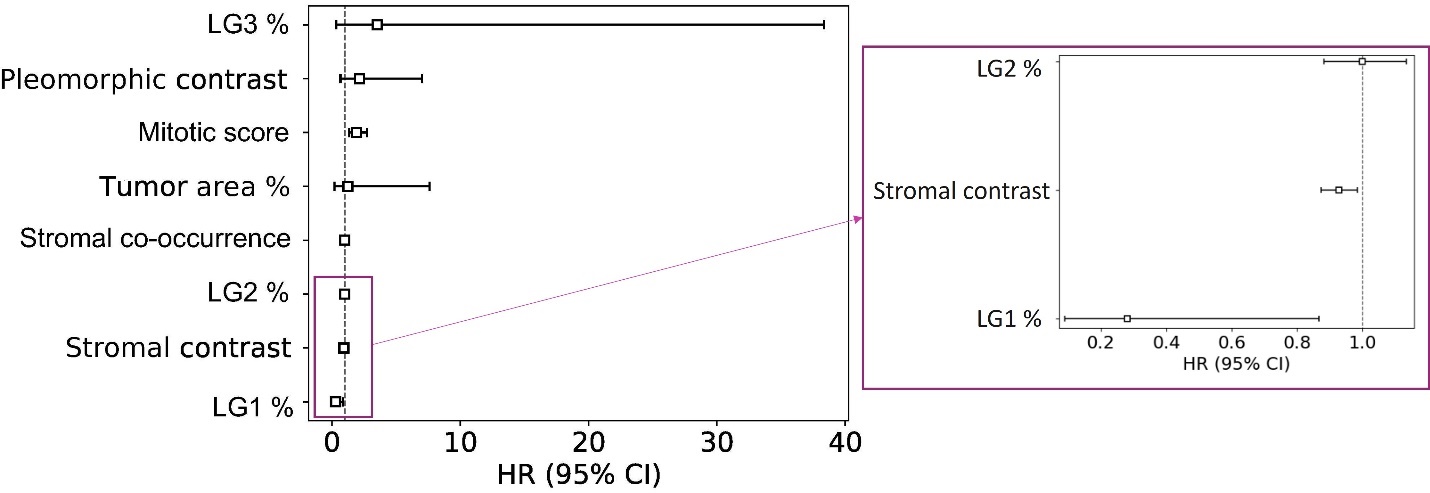


**Supplementary Figure 6 Hazard plot for BRACE marker’s components:** Hazard plot showing the contribution of BRACE marker’s components TGC(LG1 %, LG2 %, LG3 %), tumour area %, digital mitotic score, pleomorphic contrast, stromal contrast and co-occurrences of stromal nuclei patches with low density.


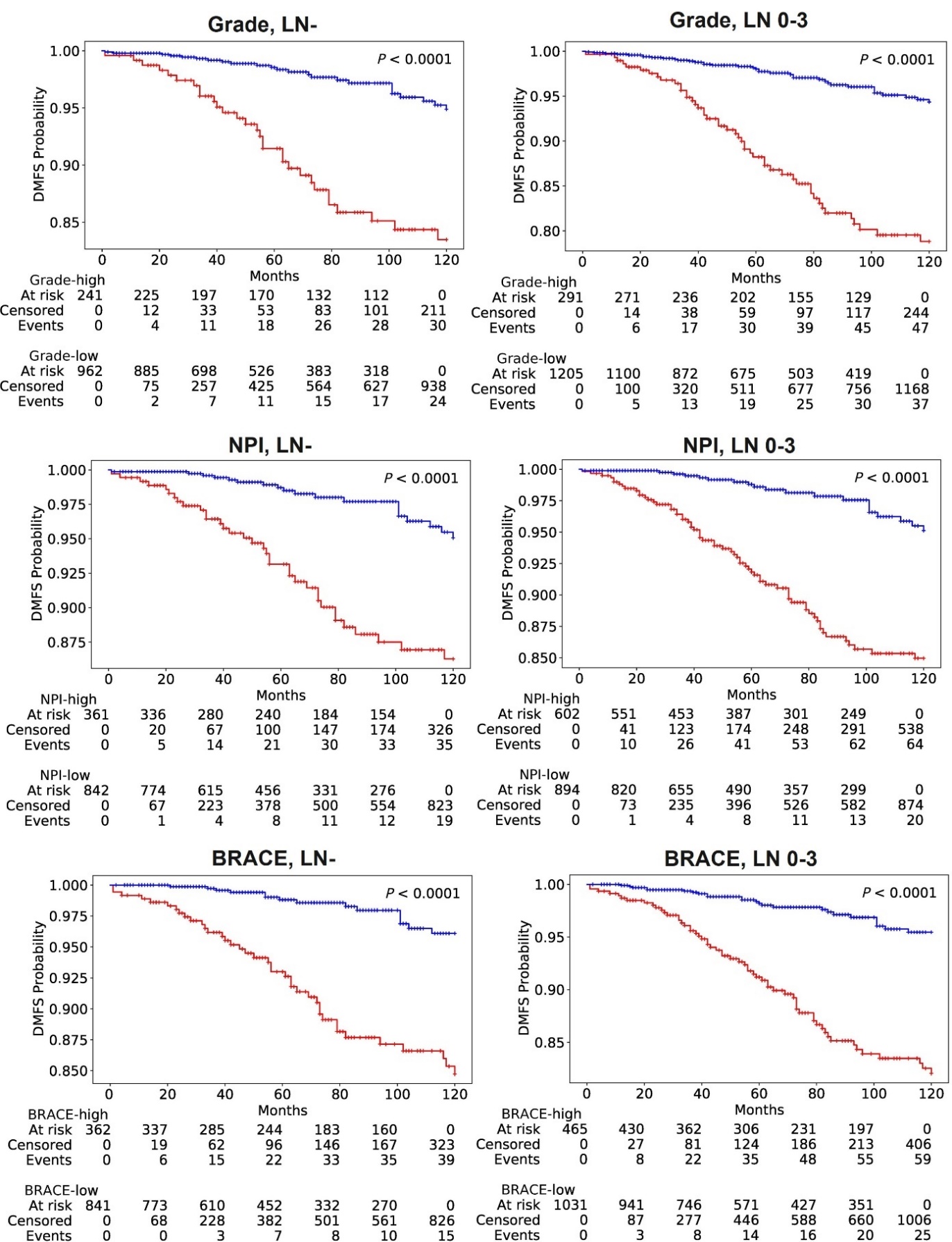


**Supplementary Figure 7 KM curves** **for DMFS**: KM curves for the high-risk (red line) and low-risk (blue line) groups of DMFS LN- (*n* = 1203) and LN 0-3 (*n* =1496 ) as stratified by BRACE marker and other clinicopathological variables on the discovery set. *P* values are for the log-rank test.


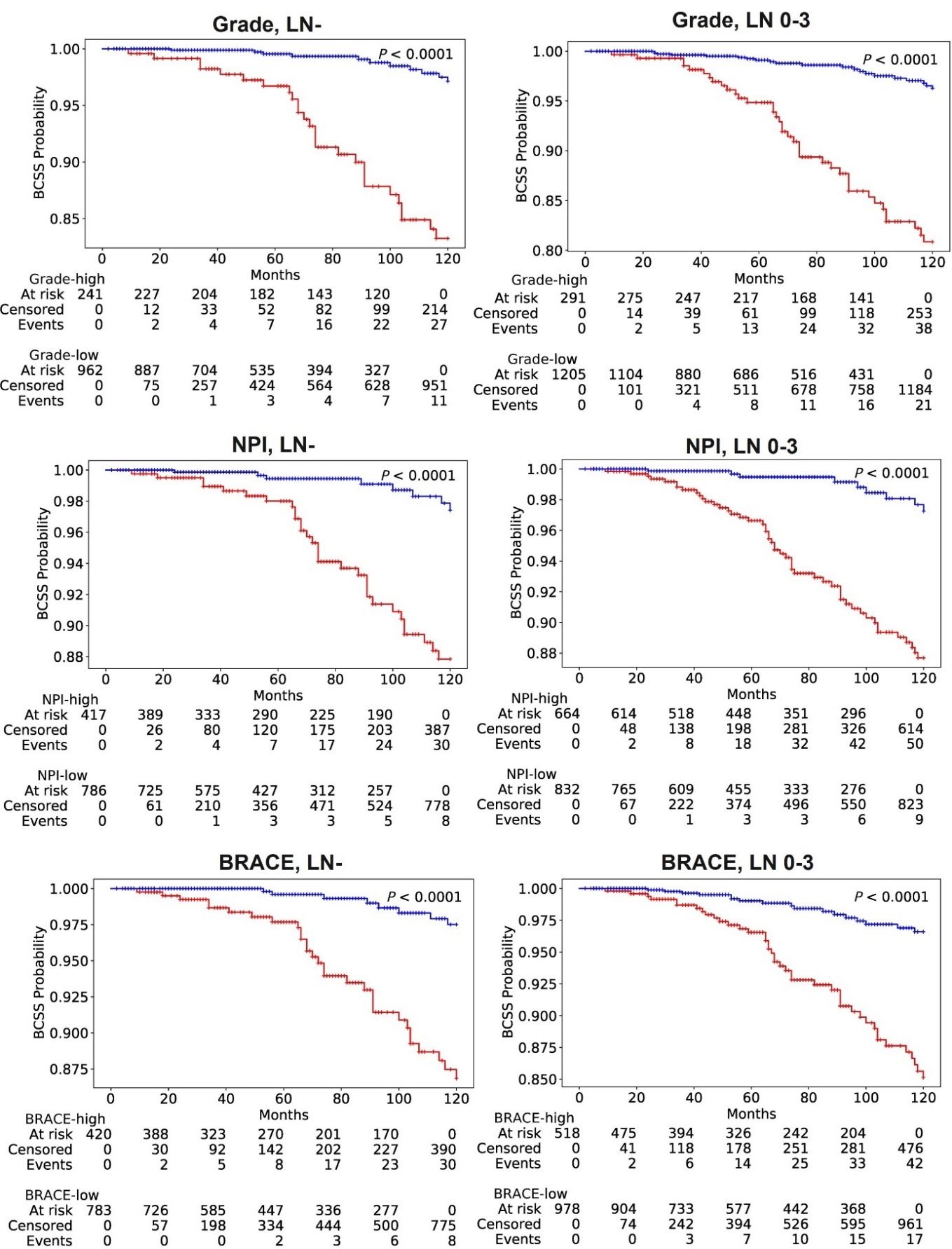


**Supplementary Figure 8 KM curves** **for BCSS**: KM curves for the high-risk (red line) and low-risk (blue line) groups of BCSS LN- (*n* = 1203) and LN 0-3 (*n* = 1496) BCSS as stratified by BRACE marker and other clinicopathological variables on the discovery set. *P* values are for the log-rank test.


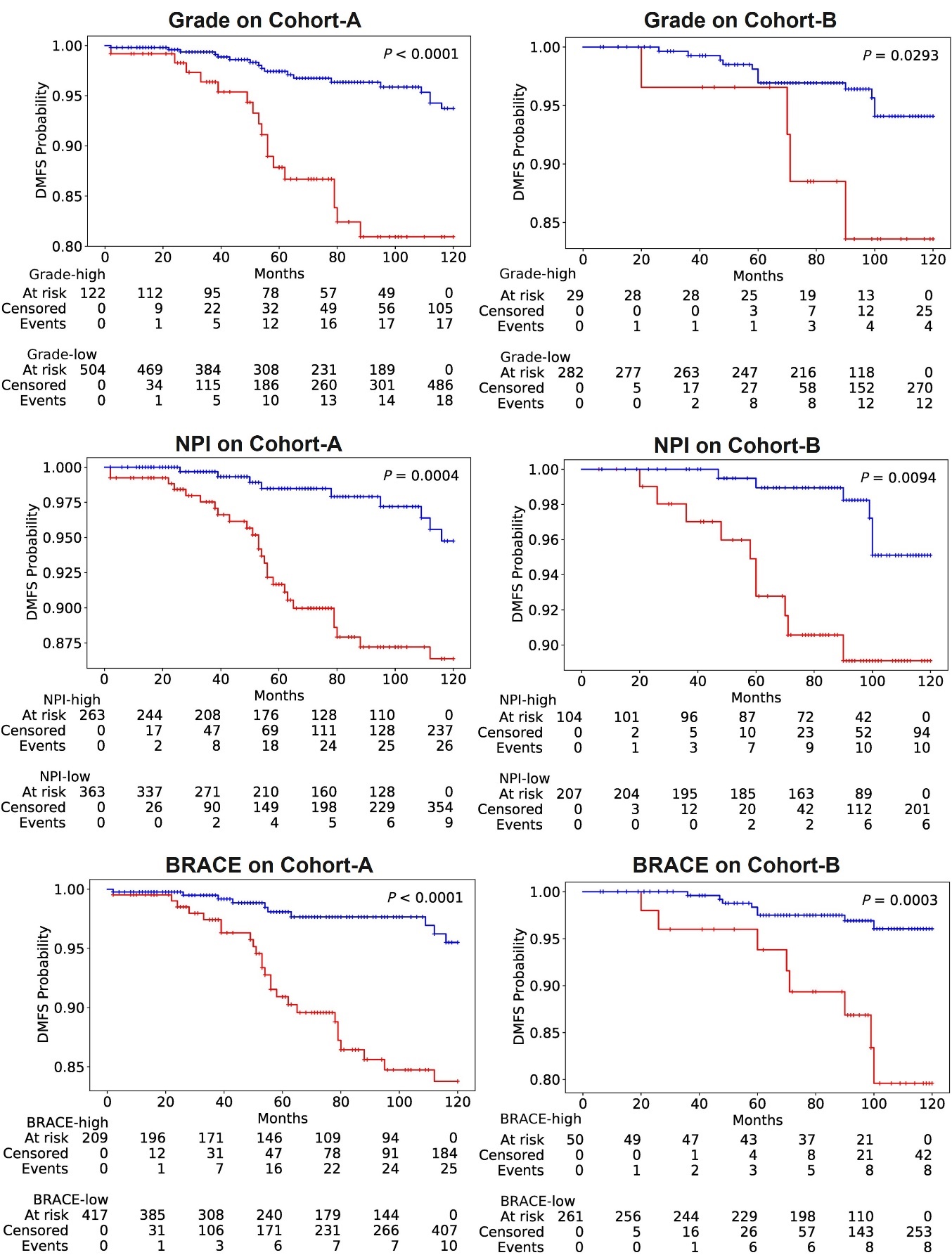


**Supplementary Figure 9 KM curves** **for LN 0-3 DMFS**: KM curves for the high-risk (red line) and low-risk (blue line) groups of DMFS LN 0-3 as stratified by BRACE marker and other clinicopathological variables on the validation sets (Cohort-A: *n* = 626; Cohort-B: *n* = 311). *P* values are for the log-rank test.


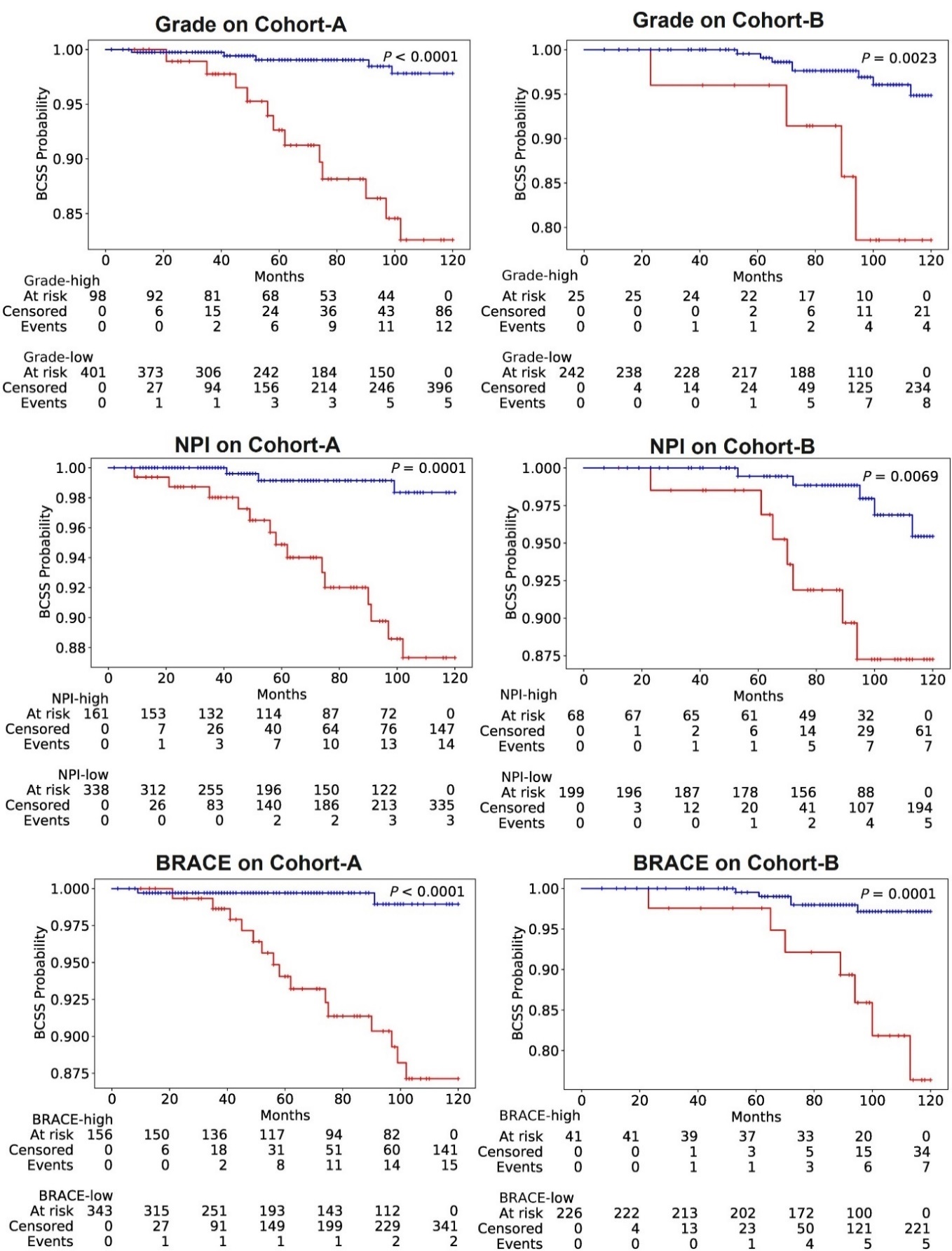


**Supplementary Figure 10 KM curves** **for LN- BCSS**: KM curves for the high-risk (red line) and low-risk (blue line) groups of BCSS LN- as stratified by BRACE marker and other clinicopathological variables on the validation sets (Cohort-A: *n* = 499; Cohort-B: *n* = 267). *P* values are for the log-rank test.


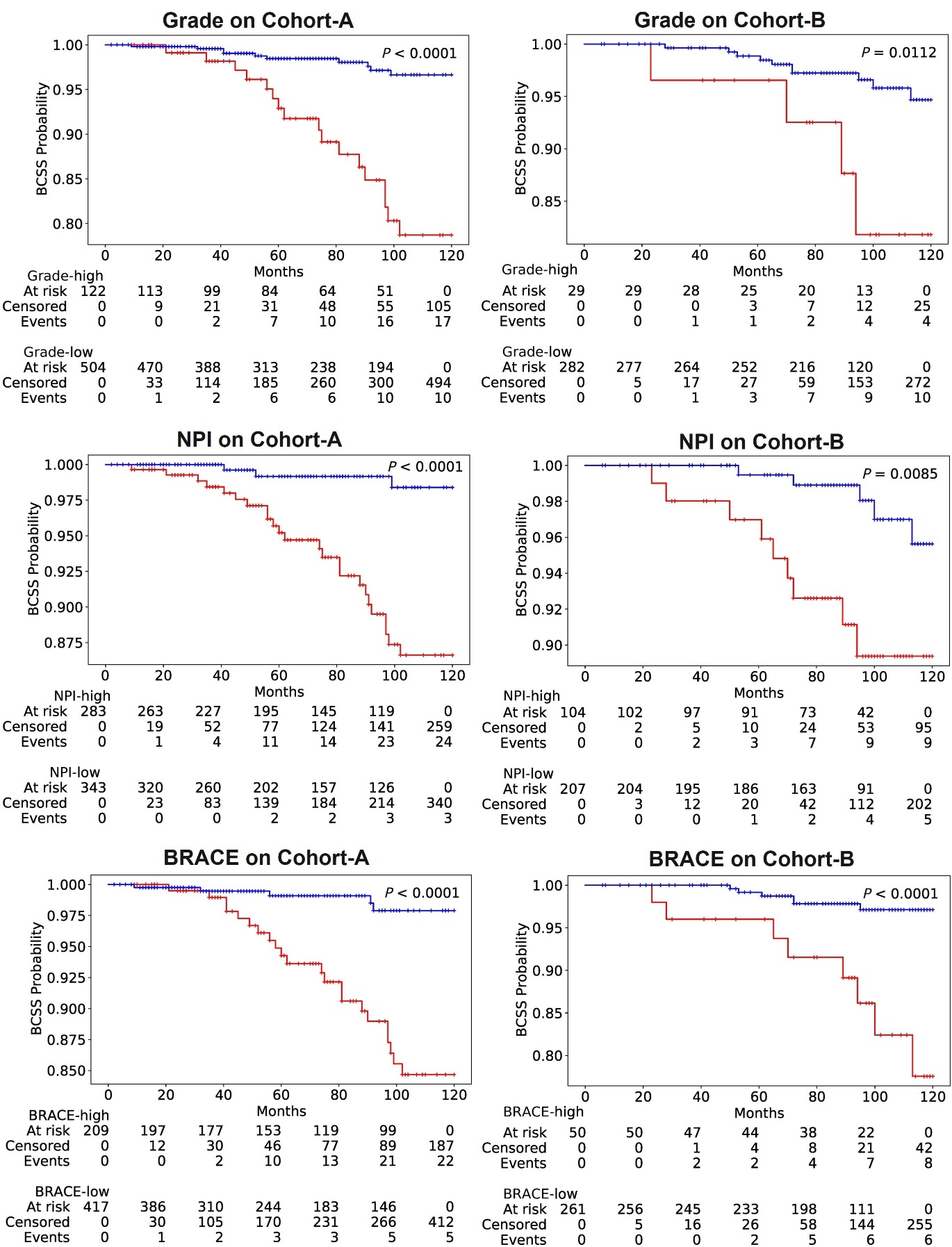


**Supplementary Figure 11 KM curves** **for LN 0-3 BCSS**: KM curves for the high-risk (red line) and low-risk (blue line) groups of BCSS LN 0-3 as stratified by BRACE marker and other clinicopathological variables on the validation sets (Cohort-A: *n* = 626; Cohort-B: *n* = 311). *P* values are for the log-rank test.


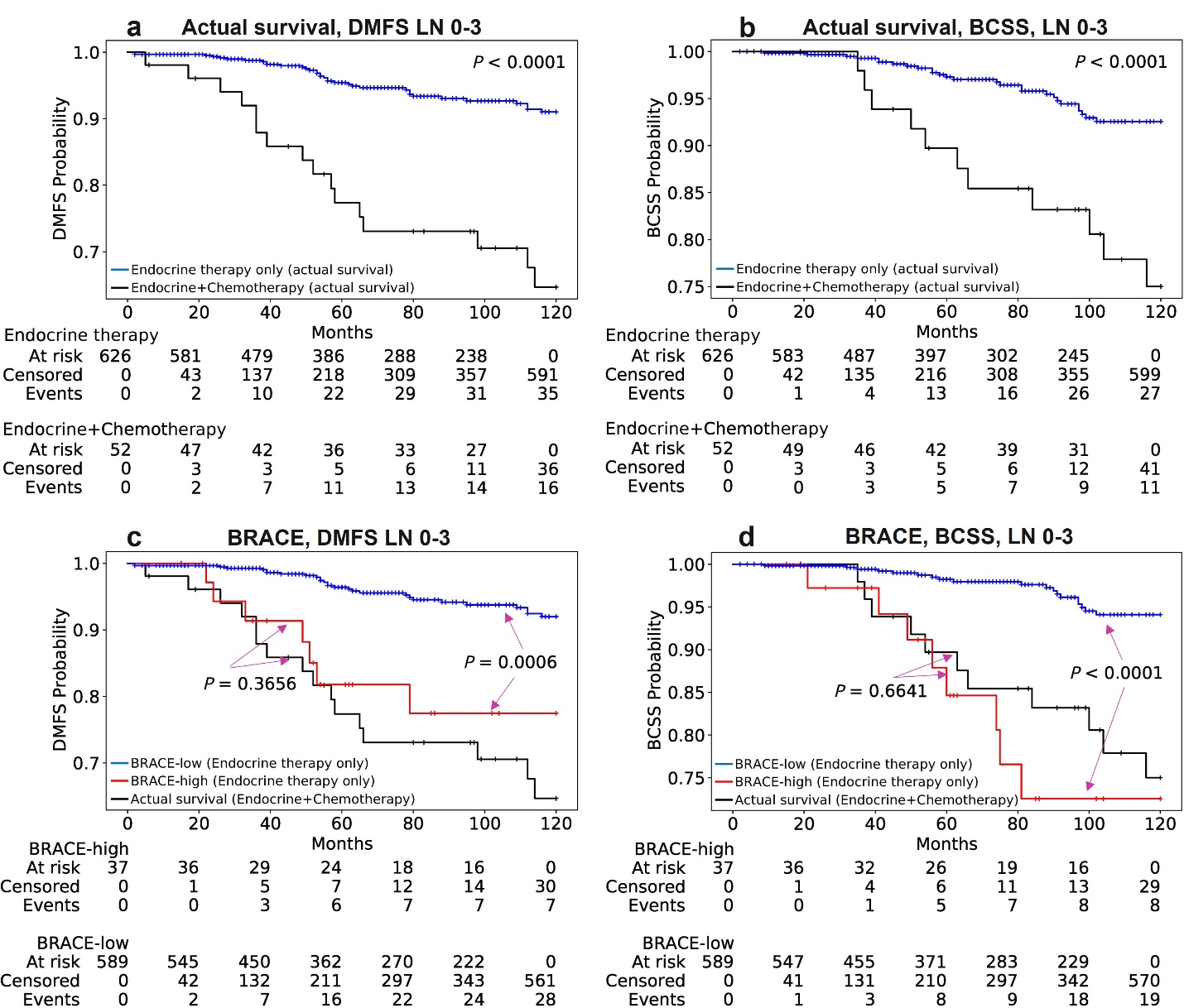


**Supplementary Figure 12 KM curves for identifying cases for chemotherapy (internal validation set):** KM curves for actual survival in endocrine therapy only treated (*n* = 626) and edocrine+chemotherapy treated (*n* = 52) patients for endpoints DMFS (a) and BCSS (b). KM curves for high-risk (red line) and low-risk (blue line) groups of LN 0-3 as stratified by BRACE marker for endpoints DMFS (c) and BCSS (d) in patients treated with endocrine therapy only in discovery Cohort-A. With appropriate cut-off BRACE identified cases which could have benefited from additional chemotherapy as shown by the overlap of the predicted high-risk curve (red line) with the actual survival curve (black line) of cases treated with chemotherapy. BRACE-high and BRACE-low represents cases identified as high- and low-risk, respectively, by BRACE marker. *P* values are for the log-rank test.


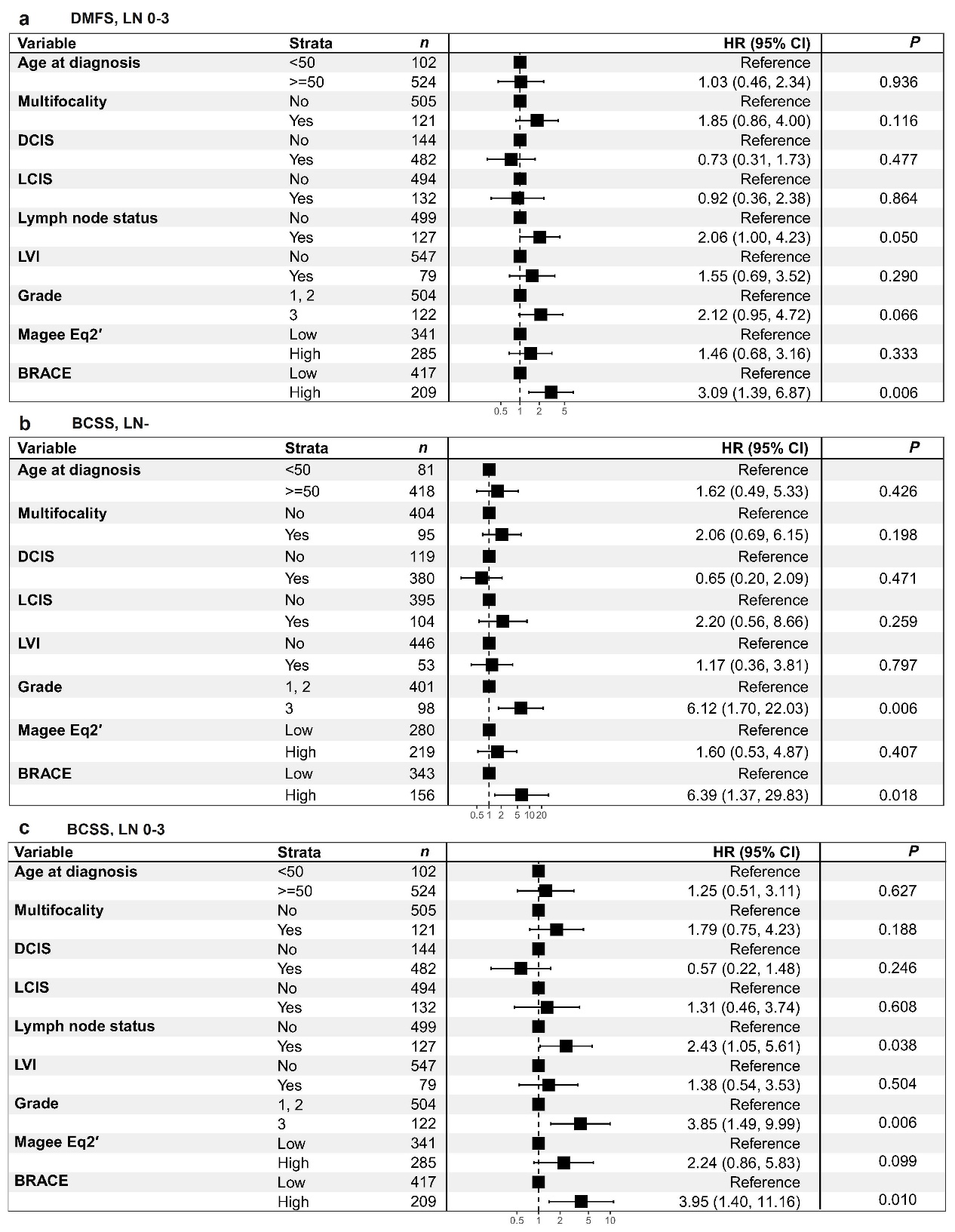


**Supplementary Figure 13 Multivariate analysis in Cohort-A:** Forest plots showing the HR with 95% confidence intervals (CI) and *P* values (of the log-rank test) for BRACE marker when adjusted for other clinicopathological variables on internal validation set of Cohort-A. (a) DMFS (LN 0-3) (*n* = 626), (b), BCSS (LN-) (*n* = 499) and (c) BCSS (LN 0-3) (*n* = 626).


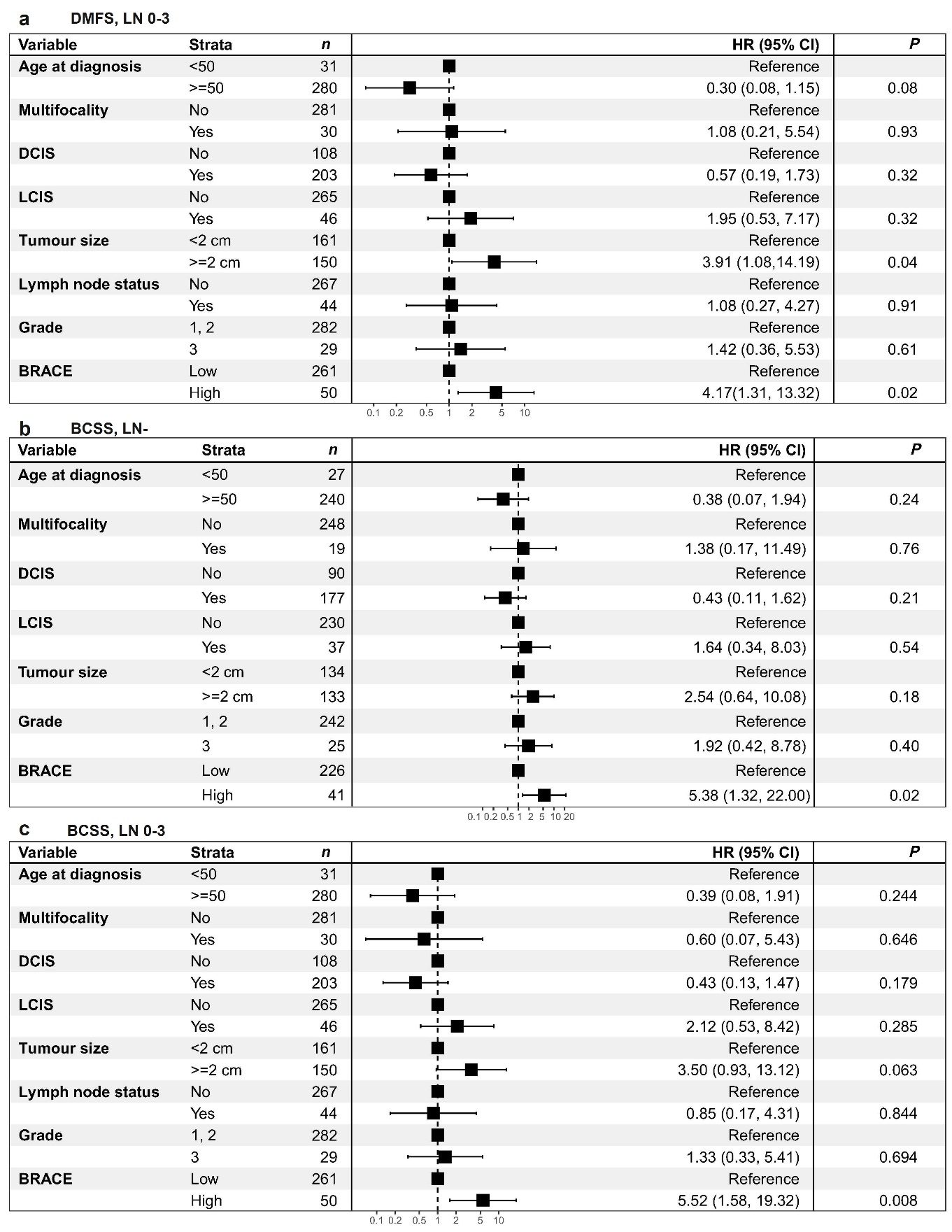


**Supplementary Figure 14 Multivariate analysis in Cohort-B :** Forest plots showing the HR with 95% confidence intervals (CI) and *P* values (of the log-rank test) for BRACE marker when adjusted for other clinicopathological variables on external validation set. (a) DMFS (LN 0-3) (*n* = 311), (b), BCSS (LN-) (*n* = 267) and (c) BCSS (LN 0-3) (*n* = 311).


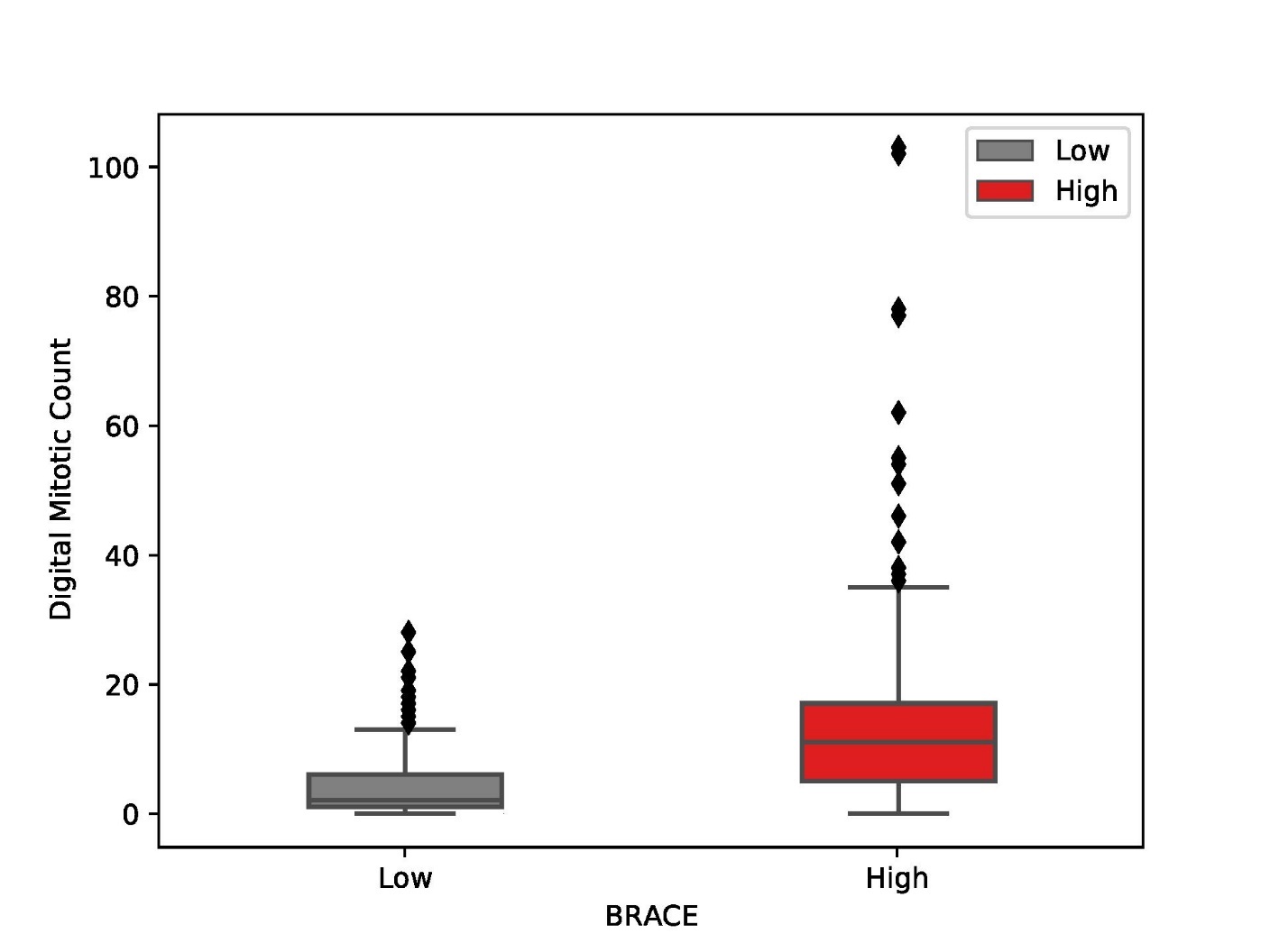


**Supplementary Figure 15 Distribution of digital mitotic count in BRACE-low vs BRACE-high groups:** Digital Mitotic Count is the number of mitoses detected by the DL model in ROIs of size 5600 × 5600 pixels at 40× selected based on high tumour cellularity and eccentricity. Plots are for DMFS (LN 0-3) of internal validation set (*n* = 626) with BRACE-low-risk (*n* = 209) and BRACE-high-risk (n = 417). The upper and lower hinges correspond to the 75th and 25th percentiles. The interior horizontal black line represents the median value. The upper whisker extends from the hinge to the largest value no further than 1.5 × IQR from the hinge; the lower whisker extends from the hinge to the smallest value at most 1.5 × IQR of the hinge.

**Supplementary Table 1** Patients’ and tumour characteristics for Cohort-A and Cohort-B. *P* value is for the Chi-square test. s.d = one standard deviation of the mean

|  |  | Cohort-A (Discovery) | | Cohort-A (Validation) | | *P* value | Cohort-B | | *P* value |
| --- | --- | --- | --- | --- | --- | --- | --- | --- | --- |
| Variable |  | Number | % | Number | % |  | Number | % |  |
| Patient age at diagnosis (years) | Mean (s.d) | 60 (11.43) | - | 60 (11.01) | - | - | 63 (9.25) | - | - |
|  | Range | 27-90 | - | 20-92 | - | - | 40-88 | - | - |
|  | < 50 | 279 | 18.65 | 102 | 16.29 | 0.197 | 31 | 9.94 | 0.0002 |
|  | ≥ 50 | 1217 | 81.35 | 524 | 83.71 |  | 281 | 90.06 |  |
| Nottingham histological grade | Grade 1 | 334 | 22.33 | 155 | 24.76 | 0.448 | 108 | 34.62 | <0.0001 |
|  | Grade 2 | 871 | 58.22 | 349 | 55.75 |  | 175 | 56.09 |  |
|  | Grade 3 | 291 | 19.45 | 122 | 19.49 |  | 29 | 9.29 |  |
| Nuclear pleomorphic score | 1 | 9 | 0.60 | 9 | 1.44 | 0.060 | 2 | 0.64 | 0.0002 |
|  | 2 | 760 | 50.80 | 336 | 53.67 |  | 198 | 63.46 |  |
|  | 3 | 727 | 48.60 | 281 | 44.89 |  | 112 | 35.90 |  |
| Tubule formation score | 1 | 142 | 9.49 | 69 | 11.02 | 0.544 | 54 | 17.31 | <0.0001 |
|  | 2 | 461 | 30.82 | 193 | 30.83 |  | 125 | 40.06 |  |
|  | 3 | 893 | 59.69 | 364 | 58.15 |  | 133 | 42.63 |  |
| Mitotic score | 1 | 1104 | 73.80 | 454 | 72.52 | 0.621 | 243 | 77.88 | 0.165 |
|  | 2 | 219 | 14.64 | 102 | 16.29 |  | 44 | 14.09 |  |
|  | 3 | 173 | 11.56 | 70 | 11.18 |  | 25 | 8.01 |  |
| Tumor size (cm) | Mean (SD) | 1.65 (0.90) | - | 1.71 (0.86) | - | - | 1.89 (1.05) | - | - |
|  | Range | 0.15-10.0 | - | 0.4-7.0 | - | - | 0.2-7.8 | - | - |
|  | < 2 | 1096 | 73.26 | 440 | 70.29 | 0.162 | 184 | 58.97 | <0.0001 |
|  | ≥ 2 | 400 | 26.74 | 186 | 29.71 |  | 128 | 41.03 |  |
| Positive lymph nodes | 0 | 1203 | 80.41 | 449 | 79.71 | 0.212 | 268 | 85.91 | 0.024 |
|  | 1-3 | 293 | 19.59 | 127 | 20.29 |  | 44 | 14.09 |  |
| Histological tumor type | Not otherwise specified (NST) | 853 | 57.02 | 371 | 59.27 | 0.613 | 221 | 70.83 | <0.0001 |
|  | Lobular | 200 | 13.37 | 73 | 11.66 |  | 48 | 15.38 |  |
|  | Other special types | 83 | 5.55 | 38 | 6.07 |  | 26 | 8.33 |  |
|  | NST mixed | 360 | 24.06 | 144 | 23.0 |  | 17 | 5.45 |  |
| Nottingham Prognostic Index (NPI) | Good (<=3.4) | 880 | 58.82 | 354 | 56.55 | 0.591 | 203 | 65.06 | 0.089 |
|  | Moderate (>3.5 and <=5.4) | 588 | 39.30 | 261 | 41.69 |  | 106 | 33.97 |  |
|  | Poor (>5.4) | 28 | 1.87 | 11 | 1.76 |  | 3 | 0.96 |  |

**Supplementary Table 2:** Representative image-based features in different categories extracted for development of BRACE marker. H: high means above a threshold, L: low means below a threshold, where the thresholds were based on the discovery sets. T: tumour, S: stroma, I: immune

| **Category** | **Image-based features** | **Description** |
| --- | --- | --- |
| **a) Tumour morphology** | **LG1-3%** | Percentage of digital local grade 1, 2 and 3 |
|  | **LP1-3%** | Percentage of digital local pleomorphism 1, 2 and 3 |
|  | **LG and LP ratios** | Ratios of different LG and LP |
|  | **Tumour nuclei size** | Minimum, maximum, mean and standard deviation of tumour nuclei size |
|  | **Tumour nuclei eccentricity** | Minimum, maximum, mean and standard deviation of tumour nuclei eccentricity |
|  | **Tumour nuclei major/minor axis** | Minimum, maximum, mean and standard deviation of tumour nuclei major and minor axes |
| **b) Tumour stroma relationship.** | **ST ratio (surface area)** | TAS patches* / (TAS patches + tumour patches) ×100 |
|  | **ST ratio (mean count)** | Mean of stromal cell count / mean of tumour cell count across all the patches in a WSI. |
|  | **ST ratio H-H co-occurrence score** | H-H STR co-occurrence / (H-H STR co-occurrence + H-L STR cooccurrence) |
|  | **ST ratio H-L co-occurrence score** | H-L STR co-occurrence / (H-H STR co-occurrence + H-L STR cooccurrence) |
|  | **% L S density patches** | Percentage of stroma patches with stroma density below or equal to the mean threshold for the individual WSI |
|  | **% H S density patches** | Percentage of stroma patches with stroma density above the mean threshold for the individual WSI |
| **c) Tumour infiltrating lymphocytes (TILs)** | **Stromal TILs** | Number of immune cells in tumour associated stroma |
|  | **Tumoural TILs** | Number of immune cells in tumour |
|  | **Stromal TILs-stromal cells co-occurrence** | Number of times stromal TILs and stromal cells are found within certain distance to each other |
|  | **Intratumoural TILs-stromal cells co-occurrence** | Number of times tumoural TILs and stromal cells are found within certain distance to each other |
|  | **Inter-WSI I density H** | Percentage of immune patches with immune cell density above the threshold obtained from all the WSIs in the discovery cohort |
|  | **Intra-WSI I density H** | Percentage of immune patches with immune cell density above the mean threshold for the individual WSI |
| **d) Heterogeneity in terms of tumour, stroma and TILs (calculated from CM)** | **Pleomorphic contrast/dissimilarity/heterogeneity** | Contrast/dissimilarity/heterogeneity in terms of predicted local pleomorphism |
|  | **Stromal contrast/dissimilarity/heterogeneity** | Local variations in terms of stromal cell densities |
|  | **iTILs contrast/dissimilarity/heterogeneity** | Local variations in terms of immune cells in tumour area |
|  | **sTILs contrast/dissimilarity/heterogeneity** | Local variations in terms of immune cells in TAS |
|  | **ST ratio contrast/dissimilarity/heterogeneity** | Contrast between high ST ratio patches and low ST ratio patches. |
|  | **Inter-WSI T density H** | Percentage of T patches with tumor cell density above the mean threshold obtained from all the WSIs in the discovery cohort |
|  | **Intra-WSI T density H** | Percentage of T patches with tumor cell density above the mean threshold for the individual WSI |
| **e) Mitotic activity** | **Mitotic count** | Number of mitotic figures in one ROI |
|  | **Mitotic score** | Mitotic score discretised into 1, 2, and 3 at different thresholds of mitotic cell counts |
|  | **Weighted mitotic count** | Sum of the probabilities of the detected mitoses |
|  | **Mitotic index** | Ratio between mitotic count to the tumour cells in one ROI |
| **f) Different nuclei** | **Counts** | Counts of tumour, normal, immune, and stomal nuclei |
|  | **Ratios** | Ratios of different nuclei e.g., tumour/normal, stomal/immune |
|  | **Percentages** | Percentage of different nuclei |

* Standard patch size = 62 µm

**Supplementary Table 3 Training and validation of the models.** For discovery, internal validation and external validation sets see Figure S1. ROC-AUC for Local grade prediction was obtained from a linear SVM trained on the proportions of local grades in each WSI against the clinical grade as the ground truth. For feature generation, all the models were applied to the whole slide instead of ROI. x ± sd for the metric represents one standard deviation of the mean (mean ± standard).

| **Module** | **Metric** | | | **Subset** | **ROI size** |
| --- | --- | --- | --- | --- | --- |
| **DCIS filter** | F1-score | | | 5-folds cross-validation on annotated tiles (*n*=13, 981) of size 1024 × 1024 pixels extracted from ROIs in the discovery set | Variable size box drawn at 5× magnification to cover a visual field |
|  | Tumour | DCIS | |  |  |
|  | 0.71±0.03 | 0.90±0.01 | |  |  |
| **Region segmentation** | Dice coefficient | | | Holdout validation (trained on annotated ROIs (*n*=193) ^3^ in the discovery set, validated on ROIs (*n*=48) in the internal validation set) | Variable size box drawn at 5× magnification to cover a visual field |
|  | Stroma | Other | |  |  |
|  | 0.76 | 0.69 | |  |  |
| **Nuclei classification (Tumour Detector)** | F1-score | | | 3-folds cross-validation on ROIs (*n*=83) ^3^ in the discovery set | Variable size box drawn at 20× magnification to cover a visual field |
|  | Immune | Tumour | Connective |  |  |
|  | 0.82±0.06 | 0.92±0.02 | 0.81±0.03 |  |  |
| **Local grade prediction** | ROC-AUC | | | 3-folds cross-validation on ROIs (1 per WSI) from discovery set | 5600 × 5600 pixels at 40× magnification |
|  | 0.83±0.01 | | |  |  |
| **Survival analysis** | p-value, C-Index, HR | | | 3-fold cross-validation on the discovery set, validated on the internal and external validation sets | NA |

**Supplementary Table 4 Nuclei classification.** Three folds cross-validation results of nuclei classification using HoVer-Net (pretrained on PanNuke-Breast) (M1), Inception Resnet v2^1^ (M2), SC-CNN^2^ (M3), Inception v3 (M4) and Tumour Detector (M5 i.e.,Hover-Net fine-tuned on Cohort-A). Pr=Precision, Re=Recall. x ± sd for precision, recall and F1 represents one standard deviation of the mean (mean ± standard) across 3-folds.

| **Model** | | | | **Overall (Macro-average)** | | | | | | | | |
| --- | --- | --- | --- | --- | --- | --- | --- | --- | --- | --- | --- | --- |
|  |  |  |  | **Precision** | | | **Recall** | | | **F1** | | |
| **M1** | | | | 0.69 ± 0.05 | | | 0.52 ± 0.08 | | | 0.55 ± 0.08 | | |
| **M2** | | | | 0.66 ± 0.04 | | | 0.62 ± 0.07 | | | 0.61 ± 0.05 | | |
| **M3** | | | | 0.68 ± 0.01 | | | 0.68 ± 0.01 | | | 0.62 ± 0.01 | | |
| **M4** | | | | 0.69 ± 0.02 | | | 0.66 ± 0.01 | | | 0.62 ± 0.02 | | |
| **M5** | | | | 0.82 ± 0.09 | | | 0.79 ± 0.02 | | | **0.79 ± 0.07** | | |
| **Cell-wise classification results** | | | | | | | | | | | | |
| **Model** | **Tumour** | | | **Immune** | | | **Connective** | | | **Normal epithelial** | | |
|  | **Pr** | **Re** | **F1** | **Pr** | **Re** | **F1** | **Pr** | **Re** | **F1** | **Pr** | **Re** | **F1** |
| **M1** | 0.75± 0.02 | 0.95±0.02 | **0.83**±0.01 | 0.94±0.03 | 0.57±0.11 | **0.71**±0.07 | 0.88±0.06 | 0.32±0.06 | **0.47**±0.07 | 0.18±0.19 | 0.24±0.17 | **0.20**±0.18 |
| **M2** | 0.91±0.03 | 0.81± 0.02 | **0.87**±0.01 | 0.84±0.04 | 0.64±0.12 | **0.72**±0.10 | 0.82±0.07 | 0.63±0.04 | **0.71**±0.04 | 0.08± 0.06 | 0.40±0.24 | **0.13**±0.08 |
| **M3** | 0.92±0.03 | 0.71±0.09 | **0.80**±0.07 | 0.89±0.05 | 0.67±0.07 | **0.77**±0.04 | 0.81±0.04 | 0.65±0.04 | **0.73**±0.04 | 0.10±0.03 | 0.69±0.05 | **0.18**±0.06 |
| **M4** | 0.91±0.03 | 0.81±0.01 | **0.85**±0.02 | 0.91±0.03 | 0.64±0.09 | **0.75**±0.06 | 0.83±0.08 | 0.63±0.05 | **0.71**±0.01 | 0.11±0.04 | 0.55±0.08 | **0.18**±0.05 |
| **M5** | 0.92±0.01 | 0.92±0.04 | **0.92**±0.02 | 0.92±0.04 | 0.74±0.11 | **0.82**±0.06 | 0.81±0.04 | 0.81±0.03 | **0.81**±0.03 | 0.60±0.33 | 0.69±0.08 | **0.59**±0.22 |

**Supplementary Table 5 Results on discovery set:** *P* value (of the log-rank test) and C-index for the proposed BRACE marker and other clinical features for DMFS and BCSS on a subgroup of endocrine treated patients with LN- and LN 0-3 are listed. Events are censored at 10 years. x ± sd for the C-Index represents one standard deviation of the mean (mean ± standard) over 1000 bootstrap runs.

| **Cohort-A (Discovery set)** | | | |
| --- | --- | --- | --- |
| **LN: Negative, Event: DMFS** | | | |
| **Feature** | ***P* value** | **C-Index** | **HR (95% CI)** |
| **Grade** | <0.0001 | 0.71 ± 0.03 | 2.65 (1.84-3.80) |
| **NPI** | <0.0001 | 0.75 ± 0.03 | 2.34 (1.71-3.14) |
| **BRACE** | <0.0001 | 0.77 ± 0.03 | 1.47 (1.30-1.67) |
| **LN: 0-3, Event: DMFS** | | | |
| **Grade** | <0.0001 | 0.72 ± 0.02 | 2.86 (2.13-3.84) |
| **NPI** | <0.0001 | 0.77 ± 0.02 | 1.38 (1.29-1.47) |
| **BRACE** | <0.0001 | 0.76 ± 0.02 | 1.50 (1.36-1.66) |
| **LN: Negative, Event: BCSS** | | | |
| **Grade** | <0.0001 | 0.77 ± 0.02 | 2.24 (1.69-2.97) |
| **NPI** | <0.0001 | 0.79 ± 0.02 | 1.82 (1.50-2.21) |
| **BRACE** | <0.0001 | 0.80 ± 0.02 | 1.60 (1.39-1.83) |
| **LN: 0-3, Event: BCSS** | | | |
| **Grade** | <0.0001 | 0.74 ± 0.03 | 2.07 (1.67-2.57) |
| **NPI** | <0.0001 | 0.80 ± 0.03 | 1.13 (1.09-1.16) |
| **BRACE** | <0.0001 | 0.75 ± 0.03 | 1.55 (1.38-1.75) |

**Supplementary Table 6 Results for a larger set of features (*n* = 484, excluding selected features):** *P* value (of the log-rank test) and C-index for a larger set of features for DMFS and BCSS on a subgroup of endocrine treated patients with LN- and LN 0-3 on the internal validation set are listed. Events are censored at 10 years. Parameter for fitting Cox model: estimation method (Breslow), L1 (1.0), L2 (0), and penalty (0.1). In order to deal with correlated features these parameters were set different than the ones used for fitting the final model after feature selection. x ± sd for the C-Index represents one standard deviation of the mean (mean ± standard) over 1000 bootstrap runs.

| **Cohort-A (validation set)** | | |
| --- | --- | --- |
| **LN: Negative, Event: DMFS** | | |
| ***P* value** | **C-Index** | **HR (95% CI)** |
| 0.0472 | 0.68 ± 0.06 | 1.12 (9.4×10^-44^-1.33×10^43^) |
| **LN: 0-3, Event: DMFS** | | |
| 0.0507 | 0.66 ± 0.05 | 1.12 (1.0×10^-32^-1.2×10^32^) |
| **LN: Negative, Event: BCSS** | | |
| 0.0347 | 0.73 ± 0.04 | 1.13 (9.9×10^-40^-1.2×10^39^) |
| **LN: 0-3, Event: BCSS** | | |
| 0.0019 | 0.74 ± 0.05 | 1.09 (1.2×10^-26^-1.0×10^26^) |

**Supplementary Table 7 Performance of a deep learning based Baseline model:** *P* value (of the log-rank test) and C-index for a baseline model for DMFS and BCSS on a subgroup of endocrine treated patients with LN- and LN 0-3 on the internal validation set are listed. Events are censored at 10 years. Base-line features were extracted from ResNet-18 (pretrained on ImageNet) on the same set of patches as BRACE. In setting 1 (listed below as *n* = 512), 512 features were extracted from Resnet18 penultimate layer. In setting 2 (listed as *n* = 10), the 512 features were reduced to 10 based on univariate C-Index. Parameters for fitting Cox model in setting 1: estimation method (Breslow), L1 (1.0), L2 (0), and penalty (0.1). In order to deal with correlated features these parameters were set different than the ones used for fitting the final BRACE model after feature selection. Parameters for setting 2 were the same as that of BRACE final feature set. x ± sd for the C-Index represents one standard deviation of the mean (mean ± standard) over 1000 bootstrap runs.

| **Cohort-A (validation set)** | | | |
| --- | --- | --- | --- |
| **LN: Negative, Event: DMFS** | | | |
| **Feature** | ***P* value** | **C-Index** | **HR (95% CI)** |
| ***n* = 512** | 0.9808 | 0.57 ± 0.09 | 1.25 (3.9×10^-105^-3.9×10^104^) |
| ***n* = 10** | 0.4796 | 0.61 ± 0.07 | 1.17 (0.88-1.56) |
| **LN: 0-3, Event: DMFS** | | | |
| ***n* = 512** | 0.5500 | 0.55 ± 0.08 | 1.17 (2.2×10^-73^-5.9×10^72^) |
| ***n* = 10** | 0.0117 | 0.65 ± 0.05 | 1.34 (1.05-1.71) |
| **LN: Negative, Event: BCSS** | | | |
| ***n* = 512** | 0.0849 | 0.57 ± 0.07 | 1.82 (1.4×10^-299^-2.3×10^299)^ |
| ***n* = 10** | 0.1087 | 0.72 ± 0.07 | 1.38 (1.09-1.75) |
| **LN: 0-3, Event: BCSS** | | | |
| ***n* = 512** | 0.6590 | 0.57 ± 0.06 | 1.15 (7.6×10^-69^-1.8×10^68^) |
| ***n* = 10** | 0.0293 | 0.71 ± 0.05 | 1.46 (1.15-1.84) |

**Supplementary Table 8 Results comparison on two scanners:** *P* value (of the log-rank test) and C-index for the proposed BRACE marker and other clinical features for DMFS and BCSS on a subgroup of endocrine treated patients with LN- and LN 0-3 on the internal validation set are listed for cases scanned with two scanners. Events are censored at 10 years. Ratio of the number of events to the number of cases is listed against each scanner in the headers. x ± sd for the C-Index represents one standard deviation of the mean (mean ± standard) over 1000 bootstrap runs.

| **Cohort-A (Validation set)** | | | | |
| --- | --- | --- | --- | --- |
| **LN: Negative, Event: DMFS, Pannoramic (12/217), Philips (11/282)** | | | | |
| **Feature** | **Scanner** | ***P* value** | **C-Index** | **HR (95% CI)** |
| **Grade** | Pannoramic | 0.6659 | 0.66 ± 0.05 | 2.29 (1.09-4.79) |
|  | Philips | 0.2243 | 0.66 ± 0.04 | 2.05 (0.90-4.66) |
| **NPI** | Pannoramic | 0.6737 | 0.66 ± 0.06 | 1.84 (1.05-3.24) |
|  | Philips | 0.0442 | 0.69 ± 0.05 | 2.22 (1.02-4.86) |
| **BRACE** | Pannoramic | 0.2345 | 0.74 ± 0.05 | 1.69 (1.28-2.21) |
|  | Philips | 0.2614 | 0.69 ± 0.06 | 1.47 (0.94-2.31) |
| **LN: 0-3, Event: DMFS, Pannoramic (19/281), Philips (16/345)** | | | | |
| **Grade** | Pannoramic | 0.3354 | 0.68 ± 0.04 | 2.05 (1.27-3.30) |
|  | Philips | 0.0439 | 0.67 ± 0.05 | 1.89 (1.18-3.05) |
| **NPI** | Pannoramic | 0.1571 | 0.68 ± 0.06 | 1.52 (1.16-2.00) |
|  | Philips | 0.0046 | 0.74 ± 0.05 | 1.43 (1.19-1.72) |
| **BRACE** | Pannoramic | 0.4145 | 0.69 ± 0.06 | 1.47 (1.22-1.76) |
|  | Philips | 0.0076 | 0.74 ± 0.04 | 1.55 (1.19-2.01) |
| **LN: Negative, Event: BCSS, Pannoramic (10/217), Philips (7/282)** | | | | |
| **Grade** | Pannoramic | 0.1174 | 0.79 ± 0.04 | 2.77 (1.48-5.18) |
|  | Philips | 0.4734 | 0.64 ± 0.05 | 1.84 (1.02-3.31) |
| **NPI** | Pannoramic | 0.0876 | 0.78 ± 0.05 | 1.67 (1.25-2.22) |
|  | Philips | 0.0072 | 0.87 ± 0.06 | 2.58 (1.38-4.83) |
| **BRACE** | Pannoramic | 0.0011 | 0.87 ± 0.06 | 2.09 (1.50-2.89) |
|  | Philips | 0.0029 | 0.74 ± 0.06 | 1.35 (0.85-2.14) |
| **LN: 0-3, Event: BCSS, Pannoramic (15/281), Philips (12/345)** | | | | |
| **Grade** | Pannoramic | 0.0523 | 0.75 | 2.00 (1.35-2.98) |
|  | Philips | 0.1457 | 0.67 | 1.90 (1.26-2.87) |
| **NPI** | Pannoramic | 0.0176 | 0.75 | 1.48 (1.14-1.90) |
|  | Philips | 0.0003 | 0.86 | 1.47 (1.23-1.74) |
| **BRACE** | Pannoramic | 0.0010 | 0.87 | 2.02 (1.48-2.76) |
|  | Philips | 0.0299 | 0.74 | 1.35 (0.85-2.14) |

**Supplementary Table 9 Results for modified BRACE*:** *P* value (of the log-rank test) and C-index for a modified version of BRACE (denoted by BRACE*) with the addition of two clinicopathological variables (i.e., lymph node status and invasive tumour size) for DMFS and BCSS on a subgroup of endocrine treated patients with LN- and LN 0-3 on the internal validation set are listed. Events are censored at 10 years. x ± sd for the C-Index represents one standard deviation of the mean (mean ± standard) over 1000 bootstrap runs.

|  | | **Cohort-A (internal validation)** | | | **Cohort-B (external validation)** | | |
| --- | --- | --- | --- | --- | --- | --- | --- |
| **LN** | **Event** | ***P* value** | **C-Index** | **HR (95% CI)** | ***P* value** | **C-Index** | **HR (95% CI)** |
| Negative | DMFS | <0.0001 | 0.73 ± 0.06 | 1.55  (1.26-1.91) | 0.0095 | 0.75 ± 0.05 | 1.19  (0.86-1.65) |
| 0-3 | DMFS | <0.0001 | 0.74 ± 0.04 | 1.50  (1.31-1.70) | 0.0130 | 0.73 ± 0.05 | 1.28  (1.01-1.61) |
| Negative | BCSS | <0.0001 | 0.83 ± 0.04 | 1.73  (1.43-2.10) | 0.0049 | 0.76 ± 0.05 | 1.20  (0.88-1.66) |
| 0-3 | BCSS | <0.0001 | 0.80 ± 0.04 | 1.61  (1.40-1.84) | 0.0034 | 0.74 ± 0.06 | 1.30  (1.04-1.64) |

**Supplementary Table 10** Association of BRACE marker with clinicopathological parameter for DMFS in LN- cases. *P* value is for the Chi-square test.

| **Parameter** | **Cohort-A (Discovery, *n* = 1203)** | | | **Cohort-A (Validation, *n* = 499)** | | | **Cohort-B (*n* = 266)** | | |
| --- | --- | --- | --- | --- | --- | --- | --- | --- | --- |
|  | **BRACE low** | **BRACE high** | ***P* value** | **BRACE low** | **BRACE high** | ***P* value** | **BRACE low** | **BRACE high** | ***P* value** |
| **Tumour Size (in cm)** | | | | | | | | | |
| **< 2** | 625 | 293 | <0.0001 | 258 | 101 | <0.0001 | 138 | 26 | 0.016 |
| ≥ **2** | 103 | 182 |  | 52 | 88 |  | 66 | 36 |  |
| **Age at diagnosis** | | | | | | |  | | |
| **< 50** | 138 | 91 | 0.99 | 47 | 34 | 0.48 | 24 | 3 | 0.63 |
| **≥ 50** | 590 | 384 |  | 263 | 155 |  | 180 | 59 |  |
| **LVI** | | | | | | | | | |
| **No** | 685 | 411 | 0.0004 | 290 | 156 | 0.0047 | 199 | 55 | 0.076 |
| **Yes** | 43 | 64 |  | 20 | 33 |  | 5 | 7 |  |
| **Menopause** | | | | | | | | | |
| **Pre** | 187 | 110 | 0.91 | 63 | 39 | 0.99 | - | - | - |
| **Post** | 541 | 365 |  | 247 | 150 |  | - | - |  |
| **PR status** | | | | | | | | | |
| **Negative** | 101 | 111 | 0.0042 | 42 | 42 | 0.232 | 19 | 6 | 0.99 |
| **Positive** | 625 | 364 |  | 268 | 146 |  | 185 | 56 |  |
| **Grade** | | | | | | | | | |
| **1** | 245 | 27 | <0.0001 | 110 | 14 | <0.0001 | 87 | 11 | <0.0001 |
| **2** | 439 | 251 |  | 176 | 101 |  | 107 | 36 |  |
| **3** | 44 | 197 |  | 24 | 74 |  | 10 | 15 |  |
| **Mitotic score** | | | | | | | | | |
| **1** | 652 | 243 | <0.0001 | 271 | 90 | <0.0001 | 172 | 35 | 0.0018 |
| **2** | 52 | 118 |  | 24 | 52 |  | 20 | 18 |  |
| **3** | 24 | 114 |  | 15 | 47 |  | 12 | 9 |  |
| **Pleomorphism score** | | | | | | | | | |
| **1** | 6 | 2 | <0.0001 | 6 | 1 | <0.0001 | 2 | 0 | 0.003 |
| **2** | 478 | 139 |  | 207 | 67 |  | 144 | 26 |  |
| **3** | 244 | 334 |  | 97 | 121 |  | 57 | 37 |  |
| **Tubule formation score** | | | | | | | | | |
| **1** | 109 | 6 | <0.0001 | 51 | 5 | <0.0001 | 45 | 4 | 0.249 |
| **2** | 237 | 120 |  | 98 | 48 |  | 78 | 28 |  |
| **3** | 382 | 349 |  | 161 | 136 |  | 81 | 30 |  |
| **NPI** | | | | | | | | | |
| **Good (≤3.4)** | 623 | 206 | <0.0001 | 255 | 75 | <0.0001 | 163 | 30 | <0.0001 |
| **Moderate (>3.5 and ≤4.4)** | 98 | 194 |  | 46 | 78 |  | 39 | 22 |  |
| **Poor (>4.4)** | 7 | 75 |  | 9 | 36 |  | 2 | 10 |  |
| **Tumour type** | | | | | | | | | |
| **Not otherwise specified (NST)** | 367 | 328 | <0.0001 | 172 | 126 | 0.06 | 140 | 48 | 0.96 |
| **Lobular** | 117 | 56 |  | 37 | 20 |  | 18 | 5 |  |
| **Other special types** | 66 | 6 |  | 31 | 3 |  | 22 | 3 |  |
| **NST mixed** | 178 | 85 |  | 70 | 40 |  | 23 | 6 |  |

1. Szegedy, C., Ioffe, S., Vanhoucke, V. & Alemi, A. A. Inception-v4, Inception-ResNet and the Impact of Residual Connections on Learning. in *Proceedings of the Thirty-First AAAI Conference on Artificial Intelligence* 4278–4284 (AAAI Press, 2017).

2. Sirinukunwattana, K. *et al.* Locality Sensitive Deep Learning for Detection and Classification of Nuclei in Routine Colon Cancer Histology Images. *IEEE Trans. Med. Imaging* **35**, 1196–1206 (2016).

3. Wahab, N. *et al.* Semantic annotation for computational pathology: multidisciplinary experience and best practice recommendations. *J. Pathol. Clin. Res.* **8**, 116–128 (2022).
